# Patient preferences for a guided self-help programme to prevent relapse in anxiety or depression: A discrete choice experiment

**DOI:** 10.1371/journal.pone.0219588

**Published:** 2019-07-18

**Authors:** Anna D. T. Muntingh, Adriaan W. Hoogendoorn, Digna J. F. Van Schaik, Annemieke Van Straten, Elly A. Stolk, Anton J. L. M. Van Balkom, Neeltje M. Batelaan

**Affiliations:** 1 Amsterdam UMC, Vrije Universiteit, Psychiatry, Amsterdam Public Health Research Institute, The Netherlands; 2 GGZ inGeest Specialised Mental Health Care, Amsterdam, The Netherlands; 3 Faculty of Behavioural and Movement Sciences, VU University, Amsterdam, The Netherlands; 4 EuroQol Office, Rotterdam, The Netherlands; Victoria University of Wellington, NEW ZEALAND

## Abstract

**Background:**

Anxiety and depressive disorders are increasingly being viewed as chronic conditions with fluctuating symptom levels. Relapse prevention programmes are needed to increase self-management and prevent relapse. Fine-tuning relapse prevention programmes to the needs of patients may increase uptake and effectiveness.

**Materials and methods:**

A discrete choice experiment (DCE) was conducted amongst patients with a partially or fully remitted anxiety or depressive disorder. Patients were presented 20 choice tasks with two hypothetical treatment scenarios for relapse prevention, plus a “no treatment” option. Each treatment scenario was based on seven attributes of a hypothetical but realistic relapse prevention programme. Attributes considered professional contact frequency, treatment type, delivery mode, programme flexibility, a personal relapse prevention plan, time investment and effectiveness. Choice models were estimated to analyse the data.

**Results:**

A total of 109 patients with a partially or fully remitted anxiety or depressive disorder completed the DCE. Attributes with the strongest impact on choice were high effectiveness, regular contact with a professional, low time investment and the inclusion of a personal prevention plan. A high heterogeneity in preferences was observed, related to both clinical and demographic characteristics: for example, a higher number of previous treatment episodes was related to a preference for a higher frequency of contact with a professional, while younger age was related to a stronger preference for high effectiveness.

**Conclusions:**

This study using a DCE provides insights into preferences for a relapse prevention programme for anxiety and depressive disorders that can be used to guide the development of such a programme.

## Introduction

Anxiety and depressive disorders are highly prevalent and often comorbid conditions, in which relapse, recurrence and chronicity are common [1,2]. Relapse (or recurrence) is defined as meeting the diagnostic criteria for the same disorder after (a period of) recovery [3]. Recovery, or remission usually indicates that a patient has no more than minimal symptoms and diagnostic criteria for a disorder are no longer fulfilled [3]. Of the patients who remit from an anxiety disorder, 24% experience a relapse into the same disorder (also referred to as the index disorder) within four years [4]. For patients with a major depressive disorder, this figure is 38% [4]. However, these numbers rise sharply when the incidence of an anxiety or depressive disorder other than the index disorder is also counted as relapse. This means that the majority (57%) of adults who remit from an anxiety or depressive disorder experience a recurrence of the index disorder or another anxiety or depressive disorder within four years after recovery [4]. These numbers exclude patients who suffer from a chronic disorder (having a disorder without remission for >2 years) which has been estimated at 25–57% for patients with an anxiety and/or depressive disorder [5]. Furthermore, adults who have once experienced an episode of an anxiety or depressive disorder, remain at a lower level of functioning [6] and most often experience some level of anxiety or depressive symptoms after recovery which is associated to recurrence of the disorder [7].

In order to lower relapse rates and improve the long-term course of depressive and anxiety disorders, effective and feasible relapse prevention strategies are needed [8]. For recurrent depressive disorders, several effective interventions aimed at relapse prevention exist. Apart from continuation treatment with antidepressants, mindfulness-based cognitive therapy [9] and preventive cognitive therapy [10] are the most thoroughly studied interventions that have proven to lower relapse rates. These interventions may be offered in a group or individually, and recently also guided self-help programmes for relapse prevention in depressive disorders have been developed [11,12]. A systematic review about psychological interventions for relapse prevention in depressive disorders identified 25 studies, resulting in a pooled relative risk (RR) of relapse of 0.64 (compared to care as usual) [13]. Interestingly, in another meta-analysis on relapse prevention, it was found that psychological relapse prevention programmes may have an additive effect to medication continuation treatment [14]. In contrast, for anxiety disorders there is hardly any evidence for the effectiveness of psychological relapse prevention programmes. In fact, only a handful of (mostly small) studies exist that evaluated the effect of relapse prevention in anxiety disorders and obsessive compulsive disorder, and these studies show mixed results [8,15–17]. However, the potential of relapse prevention in anxiety disorders is generally acknowledged, and guidelines [18,19] include the recommendation of (psychological) relapse prevention strategies, although these are often not clearly defined. Interestingly, to our knowledge transdiagnostic relapse prevention programmes, targeting both anxiety and depressive disorders, are not available, while this would seem a logical development considering the high comorbidity rates and diagnostic instability of these disorders. Transdiagnostic interventions seem to be effective [20], and probably as effective as disorder-specific interventions [21], at least in the acute phase of treatment.

Where effective relapse prevention programmes for depressive disorders are available and relapse prevention for anxiety disorders should be developed, implementation of these programmes is, and will continue to be a challenge [22]. Although uptake rates of relapse prevention interventions are unknown, we do know that preventive interventions for depression and anxiety suffer from low uptake rates [23,24] and this is probably similar in relapse prevention. Several factors are crucial for successful implementation, and patient preferences play a major role. Previously, a Dutch study found that patients suitable for a cognitive group therapy aimed at relapse prevention for either depression or anxiety often declined treatment, for various reasons [25]. Relevance of the intervention, presumed effect, contact with a professional, feasibility in their daily lives and delivery mode (group vs. individual treatment) all seemed to play a significant role in the patients’ choice of whether or not to engage in the relapse prevention programme. Patients generally preferred an individualised and low-intensity intervention, while having regular contacts with a mental healthcare professional. Low-intensity refers to interventions with minimal contact with a professional [18]. Costs did not seem to play a large role in the consideration of patients, probably because in the Netherlands all citizens have a compulsory health care insurance which includes mental health care costs, except for the first €385 (“own risk/deductible”, reference year 2016) spent on health care costs per year (excluding primary health care costs). Research amongst a sample of insured Dutch citizens shows that only 5% of the respondents indicated they used less health care because of their own risk [26].

Developing interventions which accommodate patients’ preferences may facilitate implementation and even effectiveness of interventions. It has been found that adjusting interventions to patient preferences leads to a greater acceptance of interventions, higher adherence and increased treatment gains [23,27].

Assessing patients’ preferences is a critical step in the process of designing an acceptable and effective relapse prevention programme for patients with remitted anxiety or depressive disorders. Discrete choice experiments (DCEs) are increasingly being used to explore patient preferences in healthcare [28,29]. In a DCE, participants are presented a set of choice tasks, consisting of alternative hypothetical treatment options. These hypothetical treatment options are described by treatment attributes, and respondents are asked to make a choice. The DCE allows for the calculation of the relative importance of treatment attributes in relation to the patient’s choice, and external validity when compared to actual choice behaviour (revealed preferences) has been demonstrated in several earlier applications [28,29]. Therefore, a DCE has the potential of giving a profound insight into the preferences of patients regarding optional treatments.

To our knowledge, a DCE about patient preferences for relapse prevention programmes for anxiety and depression has not yet been performed. In fact, DCEs are rarely performed in the field of mental healthcare. In a review about DCEs in healthcare, only two of the 114 included studies concerned mental healthcare [28]. Recently, Ride and Lancsar [30] published a study on preferences of pregnant or postnatal women for treatment of perinatal depression and anxiety using a DCE. The study indicated that in addition to the expected relevance of financial costs, treatment type also had a large impact on choice. In previous studies on patient preferences in mental healthcare, a preference for psychological treatment rather than medical treatment has been frequently mentioned [31]. Furthermore, a scoping review by Apolinário-Hagen and colleagues focusing on public acceptability of e-mental health interventions showed that people generally prefer therapist-assisted interventions over unguided internet treatment [32].

The aim of this study is to elicit patient preferences for a guided self-help individual relapse prevention programme using a DCE. Guided self-help was chosen as treatment modality because it seems to match the preferences of patients for an individual, low-intensity and effective treatment programme for relapse prevention [25]. This study examines heterogeneity in patient preferences for the various attributes of the DCE. It is hypothesised that patients with more residual symptoms, lower functioning and higher anxiety sensitivity (and hence a higher risk of relapse) are more likely to prefer more frequent contact and are willing to invest more time in the intervention. In exploratory analyses the influence of other patient characteristics on preference will also be investigated. The results of this study may be useful for designing acceptable and effective relapse prevention programmes for patients with remitted anxiety or depressive disorders. In addition, the results may provide useful input for the application of mental health interventions in general.

## Materials and methods

### Study design

This is a cross-sectional study amongst patients with partially or fully remitted anxiety or depressive disorders using a survey including a discrete choice experiment (DCE). The paper-and-pencil survey was completed by patients with guidance from a research assistant. The following steps were taken in the process of the design and execution of this study: 1) selection of attributes, assignment of levels, and pilot testing; 2) design of the survey; 3) experimental design; 4) data collection; and 5) data analysis. The ISPOR checklist for conjoint analysis in healthcare [33] was used to adequately report this study.

### Selection of attributes and levels, and pilot testing

A DCE is composed of multiple choice tasks in which respondents are asked to choose between two (or more) alternative, hypothetical treatments. A crucial factor in designing a DCE is the identification of key characteristics (attributes) that describe the treatment alternatives in the choice tasks. The DCE used in this study was developed in several stages, as is recommended in the literature [34]. Attributes were selected based on information derived from a scoping review of the literature (PubMed), a qualitative study and additional focus groups with experts and patients. The literature search focused on reasons to engage in treatment (preventive or otherwise) for anxiety or depression and, specifically, guided self-help [23,35–40]. The previously executed (2012–2014) qualitative study included 52 patients with remitted anxiety and depressive disorders who were eligible for participation in two RCTs about a group intervention for relapse prevention for anxiety [41] or depression [42] and examined their preferences for relapse prevention programmes [25]. Interviews were conducted using a topic list, with the following initial themes: 1) knowledge of the risk of relapse and the long-term course of anxiety and depressive disorders; 2) perceived need or relevance of relapse prevention; 3) reasons to participate or to refuse the offered intervention; 4) preferences regarding relapse prevention; and 5) the perception of the “ideal” relapse prevention programme. Following current standards of qualitative research, during the process of exploration of the data important topics were added to the initial topic list, which were: 1) using antidepressant medication as relapse prevention; 2) opinions on psychoeducation about relapse rates; and 3) willingness to invest time in relapse prevention. Results of this study were thematically analysed according to the standards of qualitative research [43]. Themes that emerged from this qualitative study were: 1) relevance of the intervention (did the participant expect to learn something); 2) presumed effect; 3) contact with a professional; 4) feasibility in their daily lives (were they able to attend the sessions at that time and place, time investment); and 5) a general preference for individual therapy over group therapy. At the start of the current study two focus groups were organised, one consisting of five experts/professionals and one of three patients in which the themes derived from the qualitative interviews were discussed (see S1 File for the interview guides). These focus groups were not part of the qualitative study, but of the preparation phase in designing the DCE. A prior decision was made by the research group that the selected attributes had to be feasible and effective for (relapse) prevention. We chose to focus on psychological treatment rather than antidepressant use as a relapse prevention strategy since many patients recover without antidepressants and psychological treatment is assumed to have a superior [13] or additive effect compared to antidepressant medication alone [14]. Based on the literature, we included effectiveness, time investment and treatment type as attributes. See Table 1 for an overview of themes which were valued as most important by professionals and patients who participated in the focus groups. Following discussion in our research group, these themes were translated into a pilot DCE with six attributes 1) professional contact frequency; 2) delivery mode; 3) treatment type; 4) programme flexibility; 5) time investment; and 6) effectiveness. The levels of the attributes were determined after discussion in the research group, taking information from the literature, the qualitative interviews and the focus groups into account. Treatment type, for example, was based on existing effective programmes for (relapse) prevention in anxiety and depression [44–47].

**Table 1:** Overview of themes valued as most important by professionals and patients (focus groups).

	Professionals (N = 5)	Patients (N = 3)
Professional	Contact with a professional patients are acquainted with	Face-to-face contact with a professional patients are acquainted with
Personalised intervention	A personal relapse prevention plan as starting point of the intervention	Programme should be tuned to the individual’s needs
Professional involvement	Professional should provide positive feedback on homework assignments	-
Contact frequency	Regular contact and/or when needed	Having contact about every three/four months and /or when needed
Intervention mode	When offered online: technical support and mobile application should be available, choice between paper and online access	Choice between paper and online access
Programme flexibility	Programme should be comprehensive	Having access to different kinds of modules or exercises (fitting the need of the patient)
Content of the intervention	Content should be coherent with treatment patient received in the past	Adequate psycho-education about relapse prevention

The attribute of a personal relapse prevention plan was omitted at first, because the idea was to be able to use the aforementioned existing, effective prevention programmes as relapse prevention programmes. A read-out-loud pilot was performed, in which five patients were asked to complete the DCE thinking out loud. Patients participating in the read-out-loud pilot were interviewed regarding the comprehensiveness of questions, the number of choice sets, and the content validity of the attributes and levels, and field notes were taken. Patients were also asked about the importance of a personal relapse prevention plan, which they deemed an essential element of the DCE. Furthermore, patients completed a rating list containing 30 attributes of a hypothetical relapse prevention programme (S2 File). However, this rating list did not discriminate enough to be used as an instrument to guide decisions on modifying the attributes of the DCE. Therefore, the outcome of the read-out-loud pilot was discussed in our research group (in which clinicians are also represented) and it was decided to add the attribute of a personal prevention plan to the DCE.

**Fig 1:**
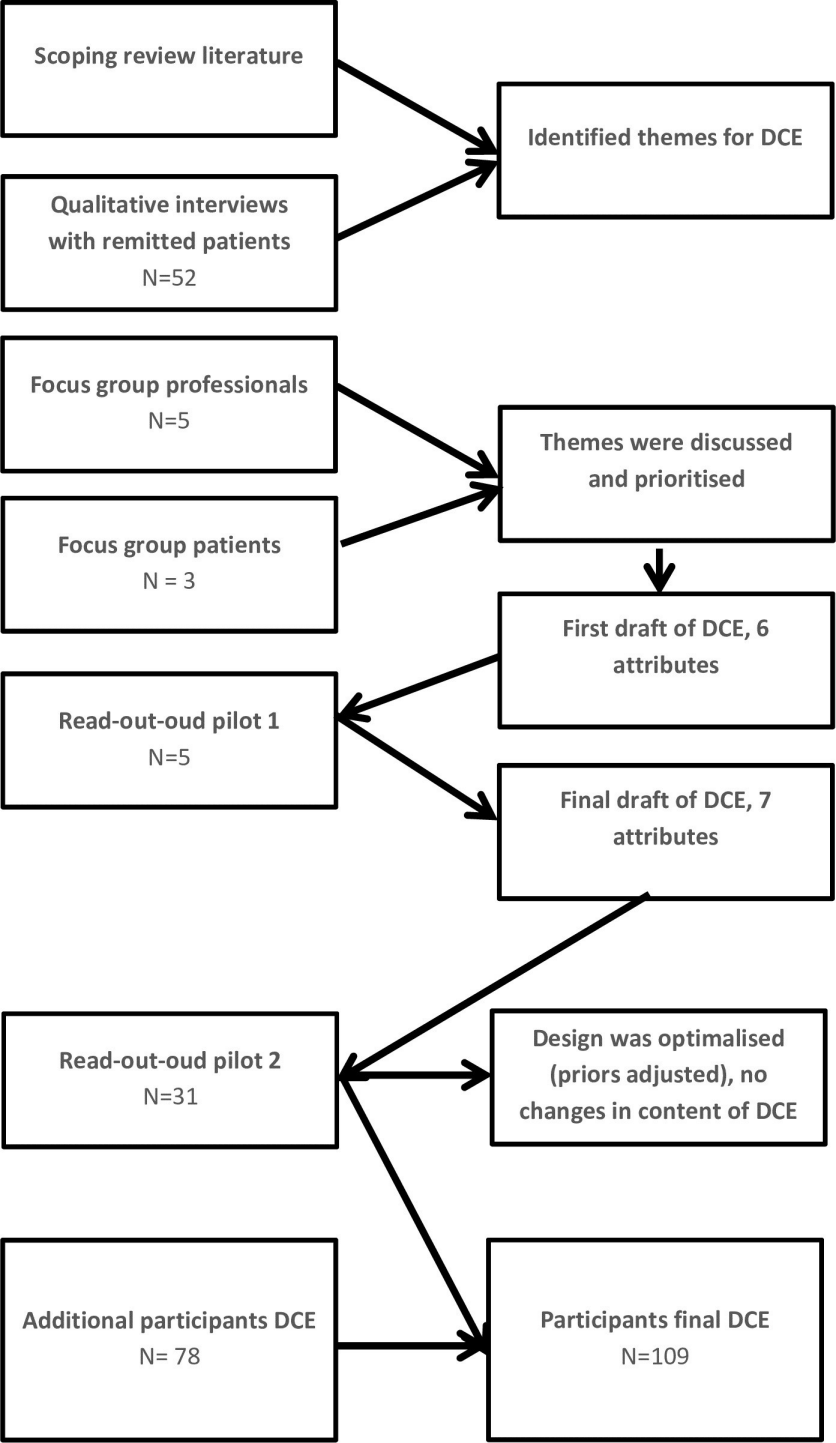
Flowchart design process of the DCE.

Few other changes were made to the survey following the read-out-loud pilot with five patients. In the explanation of attributes, screenshots of the four treatment programmes (attribute “treatment type”) were removed, as this led to choices based on the lay-out of the programme rather than the content. The screen-shots were replaced by text paragraphs explaining the content of the programme (see S3 File, pp. 7–8). Furthermore, the level of the attribute “frequency of contact” was adjusted, replacing the level “once every two months” by “once every month” to make levels more distinct for participants. Lastly, the number of choice sets was increased from 16 to 20, as this was deemed feasible by participants. Few quality checks were performed other than the pilot study. The questionnaires were administered with assistance, and engagement is normally high under these circumstances. Furthermore, the occurrence of straight lining was tested. Ultimately, the DCE consisted of 20 choice tasks, with two alternative treatments and an “opt-out” option (no treatment). The seven attributes of the treatment alternatives had two to four levels, resulting in 14 indicator variables plus the opt-out option (fifteenth indicator variable). Table 2 presents an overview of the attributes and levels of the DCE. To verify the new approach, a second read-out-loud pilot including 31 participants was performed. No new information emerged and therefore this version of the questionnaire became the final version (S3 File). These 31 participants were therefore included in the analyses of the DCE. See Fig 1 for a flowchart of the design process.

**Table 2:** Attributes and levels of the discrete choice experiment (DCE).

Attributes	Levels
Frequency of meetings with a professional	Once per month
	Once every 3 months
	Once every 6 months
	Only if you suffer a relapse
Delivery mode	App
	Website
	Book
Programme flexibility	Complete 10-week course
	Individual modules/exercises you can choose from
Treatment type	Cognitive behavioural therapy
	Problem solving therapy
	Positive psychology
	Mindfulness
Personal prevention plan	Included in intervention
	Not included in intervention
Time investment	½ hour per week
	1 hour per week
	2 hours per week
Effectiveness (relapse protection)	The risk of relapse decreases from 60% to 54%
	The risk of relapse decreases from 60% to 45%
	The risk of relapse decreases from 60% to 36%

### Design of the survey

The survey consisted of three parts, respectively presenting: 1) questions on socio-demographic, clinical and treatment-related characteristics; 2) the DCE including explanation of the attributes; and 3) self-report questionnaires about anxiety, depression and quality of life (see S3 File for parts A and B). To verify understanding of attributes, levels and the choice task, a research assistant was present who explained the study to the participant using a pre-written text, gained informed consent and answered any questions regarding the content of the attributes. Patients completed the first example choice task in read-out-loud form to verify whether the patient understood how the DCE worked.

#### Socio-demographic, clinical and treatment-related characteristics

The following socio-demographic, clinical and treatment-related characteristics were assessed based on self-report: gender, age, educational level, primary diagnosis, received treatment, treatment history, experience with self-help or online therapy, age of onset, family history of anxiety or depression, and the subjective (self-perceived) risk of relapse. The self-reported diagnosis was verified with the patient’s clinician.

#### DCE

**Fig 2:**
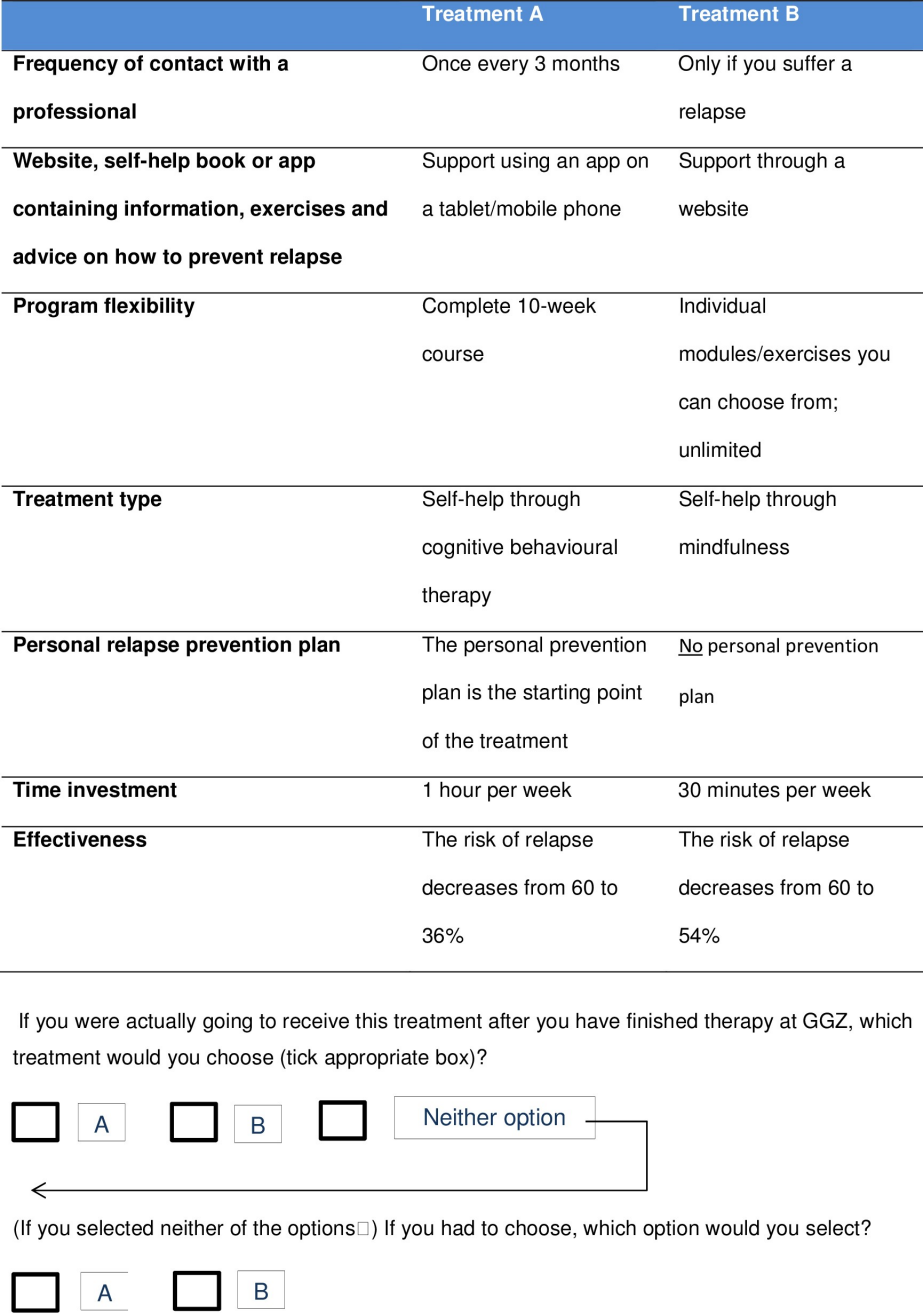
Example of a choice task in the DCE.

Before patients were presented the choice tasks, the attributes and levels of the treatment programme were described to ensure all attributes were clear to participants. See Fig 2 for an example of a choice task. Patients were asked to choose between treatment A and treatment B, but there was also an “opt-out” option (“neither”). Subsequently, they were asked what they would choose if they were obliged to (forced choice).

#### Self-report questionnaires

The survey included four self-reported questionnaires regarding symptoms of anxiety or depression and functioning which are related to the risk of relapse [48,49]: 1) the WHO Disability Assessment Schedule (WHODAS) [50]; 2) the Beck Anxiety Inventory (BAI) [51]; 3) the Inventory of Depressive Symptomatology (IDS) [52]; and 4) the Anxiety Sensitivity Index (ASI) [53].

The WHODAS is a 36-item questionnaire that measures functioning and disability during 30 days prior to the completion of the questionnaire [50]. Items are divided into six life domains: cognition, mobility, self-care, getting along with other people, life activity, and participation in society. A five-point Likert scale, ranging from 1 (no difficulties) to 5 (extreme difficulties) was used to score the items of the WHODAS. The sum score of the WHODAS was calculated by using the complex scoring method that is explained in the manual of the WHODAS, and ranges from 0 (no disability) to 100 (full disability) [50]. The BAI was used to measure anxiety [51]. The BAI is a self-report measure with 21 items with a four-point Likert scale. Sum scores range from 0 (no anxiety symptoms) to 63 (severe anxiety symptoms). The IDS is a self-report questionnaire including 30 items to score depressive symptoms [52]. The items are scored on a Likert scale from 0 to 3, yielding a sum score which ranges from 0 (no depressive symptoms) to 84 (severe depressive symptoms).

The ASI is a questionnaire that measures anxiety sensitivity, which relates to fear of anxiety-related somatic sensations [53]. The ASI includes 16 items and uses a five-point Likert scale ranging from 1 (hardly) to 5 (very much), which results in a total range of 16 to 80.

### Recruitment of participants

Recruitment took place from May 2014 to May 2016 in two outpatient mental health clinics in Amsterdam, the Netherlands. Patients were eligible if they had a primary diagnosis of an anxiety disorder according to the DSM-IV (generalized anxiety disorder, panic disorder, social phobia, simple phobia, obsessive compulsive disorder, posttraumatic stress disorder), or a unipolar depressive disorder, were remitted (partially or fully) after psychotherapeutic or pharmacological treatment based on the judgement of their clinician (a psychiatrist or clinical psychologist), and were expected to conclude treatment within the next three months. According to DSM-IV, partial remission means that patients still experience symptoms of anxiety or depression, but do not fulfil the necessary diagnostic criteria to justify a diagnosis. Patients with a remitted anxiety or depressive disorder who had recently (<6 months) concluded treatment at the outpatient clinic also received an invitation to participate in this study. A priori, we estimated that we would be able to include between 100 and 200 patients in this study using this mode of data collection. De Bekker-Grob et al. [54] indicate that this number of participants generally suffices for modelling preference data. The study protocol was approved by the Medical Ethics Review Committee of VU University Medical Centre on June 23, 2014 (reference number 2014.247).

### Data collection

Clinicians were asked to actively recruit patients who were in the end phase of treatment. Furthermore, a researcher searched the electronic medical records for patients with a primary diagnosis of an anxiety or depressive disorder who had concluded treatment in the previous six months. Subsequently, the patient’s clinician was asked to check inclusion criteria and to inform the patient about our study. Eligible patients received an information letter explaining the study and an informed consent form. All eligible patients received a telephone call from a research assistant whose task it was to explain the study and assess willingness to participate. If patients agreed to participate, they were invited for an assessment with a research assistant at the outpatient clinic. The assessment took approximately 60 minutes per patient. If patients were unwilling or unable to visit the outpatient clinic, questionnaires were completed at home with telephone assistance from the research assistant. All participants were adults without significant (current) mental health problems. Participants provided written informed consent prior to participation.

#### Experimental design

The software Ngene [55] was used to produce a D-efficient design with 20 choice sets for each participant. The efficient design approach was preferred over alternative approaches due to the sample size. Prior parameter values were specified for each fixed parameter assuming no effects other than a preference for low time investment and high effectiveness (see S4 File for the Ngene syntax). To account for uncertainty around those priors, we interrupted data collection after the first set of participants (N = 31), which is recommended to be able to estimate priors. The information from the preliminary analysis was used as input for another run using the Ngene software to create a Bayesian efficient design for the remainder of the patients. The design was constrained by excluding the combinations of maximal effectiveness with minimal time investment and vice versa. In the final design, attributes with two or four levels were in balance (10:10 or 5:5:5:5). Attributes with three levels were either distributed as 7:7:6 or 8:6:6 (see also S3 and S4 Files).

The update of the design assumes poolability. However, it is not obvious that this assumption will hold in all circumstances. When creating efficient designs, there is a trade-off between statistical efficiency and respondent efficiency. Tasks that are statistically more efficient are also expected to be more difficult for respondents and may result in different error structures. Pooling data with different error structures may lead to biased parameter estimates. Poolability was evaluated using the Swait & Louviere (SL) test [56], testing equality of the *β*-parameters except for a scale parameter that captures the differences between the *β*-parameters the two data sets. After this first test, in case of non-rejection, equality of the scale parameter would be tested in a second step. In case of rejection of the SL test, heterogeneity was further studied, in a model using all design-by-attribute-level interaction terms.

### Data analysis

Descriptive analyses about the socio-demographic and clinical variables were carried out in SPSS, version 20. The data of the DCE were analysed in Stata, version 14, using logistic models that relate the patients’ choice of treatment A, treatment B or neither (“opt-out”) to the characteristics of the treatments by means of a utility function. The utility function of the treatment involved 14 indicator variables in order to take all attributes with varying levels into account, using the first category as a reference category: professional contact frequency (four levels), delivery mode website/book/app (three levels), treatment type (four levels), programme flexibility (two levels), personal prevention plan (two levels), time investment (three levels) and effectiveness (three levels). The fifteenth indicator variable refers to the utility of the opt-out option.

The choice data were first analysed using a conditional logit model, a relatively simple model that assumes homogeneity in patients’ preferences (Model 1). The following utility function *U*_*ij*_ for individual *i* of alternative *j* consisted of a systematic component (*V*) and a random component (*ε*), and was specified as

$$U_{ij}=V_{ij}+\varepsilon_{ij}$$

where we assume that the systematic component *V*_*ij*_ is a function of the attributes of the alternative:

$$\begin{array}{c} V_{ij}=\beta_{0}D_{\text{non}-\text{treatment versus treatment}}+\beta_{11}D_{\text{personal contact frequency}\;\left(\text{pcf}\right)=\text{every }6\;\text{months}}+\beta_{12}D_{\text{pcf}=\text{every}\;3\;\text{months}\;}\\ +\beta_{13}D_{\text{pcf}=\text{every month}\;}+\beta_{21}D_{\text{delivery mode}\;\left(\text{dm}\right)=\text{website}\;}+\beta_{22}D_{\text{dm}=\text{app}}\\ +\;\beta_{3}D_{\text{programme flexibility}=\text{individual modules and exercises}}+\beta_{41}D_{\text{treatment type}\;\left(\text{tt}\right)=\text{problem solving therapy}}\\ +\beta_{42}D_{\text{tt}=\text{positive psychology}}+\beta_{43}D_{\text{tt}=\text{mindfulness}}+\beta_{5}D_{\text{personal prevention plan}=\text{included}}\\ +\beta_{61}D_{\text{time investment}\;\left(\text{ti}\right)=1\;\text{hour per week}}+\beta_{62}D_{\text{ti}=2\;\text{hours per week}}\\ +\beta_{71}D_{\text{relapse protection}\;\left(\text{rp}\right)=\;\text{decrease in risk of relapse to}\;45\text{\%}}+\beta_{72}D_{\text{rp}=\;\text{decrease in risk of relapse to}\;36\text{\%}} \end{array}$$

Equation 1: Utility function of the attributes in the DCE.

The *β*s are parameters to be estimated, the *D*-variables are dummy (or indicator) variables of characteristics of the treatment. The Alternative Specific Constant (ASC/*β*_0_) indicates the utility of no treatment over treatment and is only in the utility function for the opt-out choice, in which case it is the only parameter in the utility function. For the remaining *D*-variables, *pcf* denotes incidental professional contact as the reference category, *dm* denotes delivery mode, taking self-help book as reference, *pf* denotes programme flexibility, taking a fixed programme as reference, *tt* denotes treatment type, taking cognitive behavioural therapy (CBT) as reference, *ppp* denotes personal prevention plan, taking not included in intervention as reference, *ti* denotes time investment, taking a half hour per week as reference, and *rp* denotes relapse protection, taking the risk of relapse decreases from 60% to 54% as reference.

Next, we explored heterogeneity by adding attribute-by-patient-characteristics interaction terms to the conditional logit model. Because there is virtually no information available on which patient characteristics are related to preferences in mental health care, these analyses were exploratory, based on statistical significance. In a first analysis we re-estimated the conditional logit model several times with a single interaction added (i.e. for each combination of eight sets of *β*-parameters*19 patient characteristics). To summarize these results in a single conditional logit model, we added all highly significant (i.e. p<0.001) interactions to a backward-stepwise method of estimating the conditional logit model (Model 2). All available socio-demographic, clinical and treatment-related characteristics were tested for interaction with the attributes: age, gender, education level, type of disorder (anxiety or depressive disorder), previous type of treatment (CBT, interpersonal therapy, medication, e-health, self-help (guided or unguided)), history of psychiatric treatment, number of previous treatment episodes, age of onset, family history of anxiety or depression, anxiety severity (BAI), depression severity (IDS-SR), anxiety sensitivity (ASI), and quality of life (WHODAS-36).

Subsequently, we estimated a mixed logit model, which is the current standard, and which allows for individual heterogeneity in preferences. In the mixed logit model, the utility parameters *β* may differ across patients according to a normal distribution for parameters whose distribution parameter was estimated. The mixed logit model contained the interaction terms found in the analysis from Model 2 as non-random coefficients, while all attributes and the Alternative Specific Constant were supposed random with normal distribution (Model 3).

The relative importance of each attribute on patients’ choice can be deduced from the model by studying the estimated parameters and their standard errors. To facilitate the interpretation, we used predicted probability analysis [57] as applied in [30]. We selected somewhat arbitrarily a base treatment defined by the following (base) levels: a complete 10-week course, using a self-help book, based on cognitive behavioural therapy, without personal prevention plan, having contact with a professional only if suffering a relapse, assuming time investment of ½ hour per week and with a risk of relapse decreased to 54%–and evaluated the probability that this treatment would be chosen over non-treatment. Next, we evaluated the probability of an alternative treatment, varying the alternative treatment systematically over the levels of each attribute, and computing the relative increase in probability of selecting the alternative, thus obtaining a graphical display of the importance of all attribute levels.

Lastly, because a high anxiety or depression score was not an exclusion criterion, and the inclusion of patients with severe anxiety or depression may affect the results of the analyses, a sensitivity analysis was performed. In a conditional logit model, patients with a score in the category “severe” or “very severe” on the BAI (anxiety) or IDS (depression) were excluded to see whether this influenced the results compared to the original conditional logit model.

## Results

**Fig 3:**
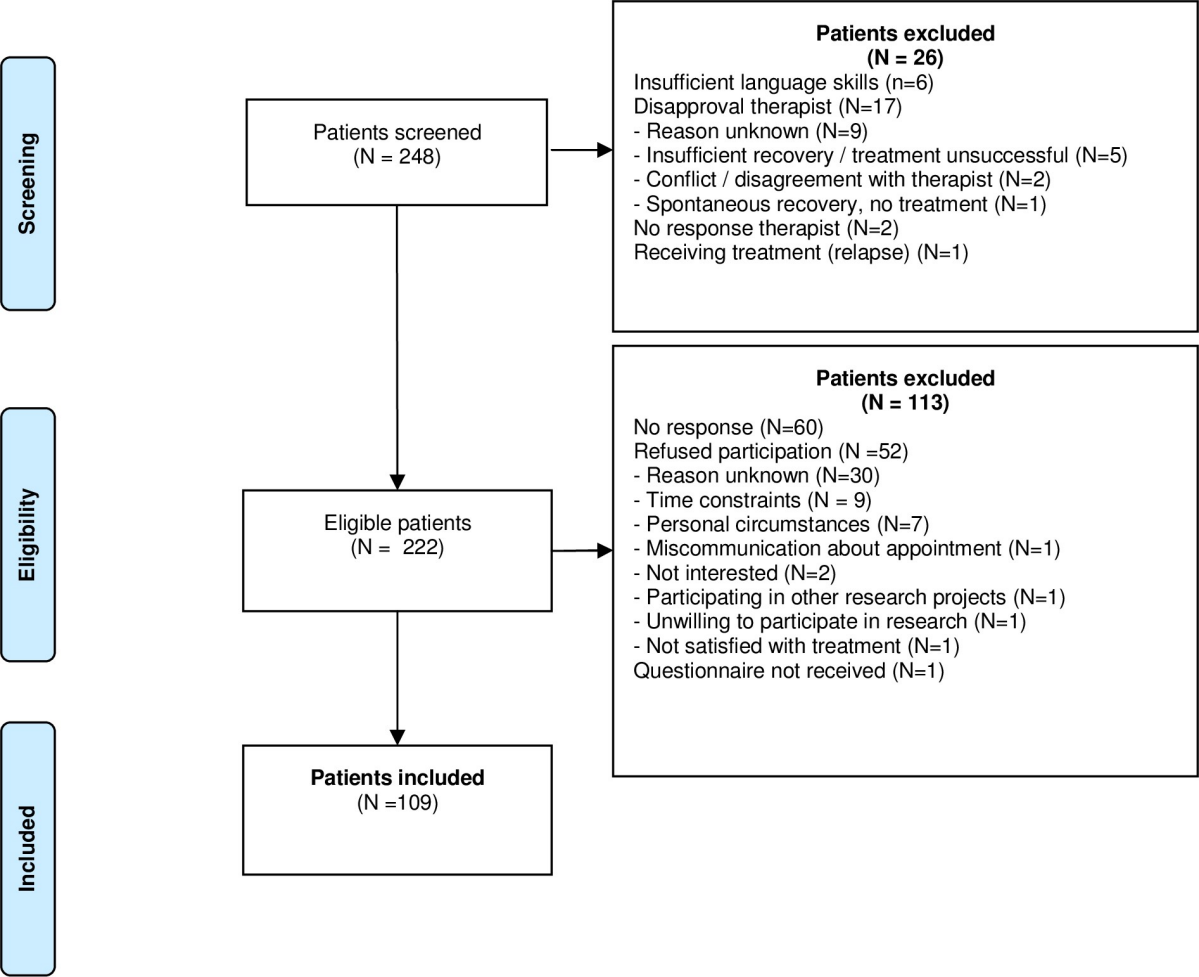
Flowchart of participants.

A total of 248 patients were screened for eligibility. Of these, 26 were excluded because they did not fulfil the inclusion criteria (N = 13), or because the therapist did not approach the patient (N = 13). Another 54 patients declined participation and 60 patients were excluded because we were unable to contact them. Ultimately, 109 patients completed the DCE: 31 patients using a D-efficient design using assumed priors for the attributes effectiveness and time investment, and 78 using an adapted Bayesian efficient design (S4 File). Fig 3 shows the flowchart of participants.

### Characteristics of participants

Table 3 describes the baseline characteristics of the participants. Of the participants, 64% were female, with a mean age of 41.3 years (standard deviation (SD) 12.7, range 21 to 69). The majority of participants had been treated previously for anxiety or depression (70%). According to the BAI and IDS-SR scores, depressive and anxiety symptoms were mild on average (BAI mean 11.1, SD 9.5; IDS-SR mean 18.1, SD 11.5). Most participants had received cognitive behavioural therapy (85%) and/or pharmacotherapy (54%). Experience with self-help or internet treatment was low (19 and 6% respectively). Participants estimated their risk of relapse at 45% (SD 23.1). The mean level of disability according to the WHODAS-2.0 (WHODAS-36 mean score 19.5, SD 13.4) was higher than in the general population (percentile 78.4–82.7), but lower than for patients with current depressive or anxiety disorders [6].

**Table 3:** Socio-demographic, clinical and treatment-related characteristics of participants (N = 109).

(N = 109)	N (%)	M (SD)
**Socio-demographic characteristics**		
Age		41.3 (12.7)
Gender		
- Female	70 (64.2%)	
Educational level		
- Low (max. 13 years of education)	16 (15.0%)	
- Intermediate (≥ 13 years of education)	35 (32.7%)	
- High (≥ 15 years of education)	56 (52.3%)	
**Clinical characteristics**		
Self-reported focus of treatment		
- Anxiety	42 (38.5%)	
- Depression	28 (25.7%)	
- Both anxiety and depression	39 (35.8%)	
Self-reported focus of treatment		
- Unipolar depressive disorder	44 (41.1%)	
- Dysthymia	1 (1.00%)	
- Panic disorder (with/without agoraphobia)	18 (16.8%)	
- Generalized anxiety disorder	11 (10.3%)	
- Social anxiety disorder	8 (7.4%)	
- Obsessive compulsive disorder	17 (15.9%)	
- Post-traumatic stress disorder	3 (2.8%)	
- Specific phobia	1 (1.0%)	
- Anxiety disorder NOS	4 (3.7%)	
Age of onset		27.3 (12.7)
Family member (first grade) with anxiety or depression	57 (52.3%)	
IDS severity score		18.1 (11.5)
IDS severity category		
- None (0–13)	48 (45.3%)	
- Mild (14–25)	29 (27.4%)	
- Moderate (26–38)	23 (21.7%)	
- Severe (39–48)	4 (3.8%)	
- Very severe (≥49)	2 (1.9%)	
BAI severity score		11.1 (9.5)
BAI severity category		
- Normal (0–9)	62 (58.5%)	
- Mild (10–18)	27 (25.5%)	
- Moderate (18–29)	9 (8.5%)	
- Severe (≥30)	8 (7.5%)	
ASI		11.0 (10.2)
WHODAS-36		19.5 (13.4)
**Treatment-related characteristics**		
Self-reported focus of treatment		
- Unipolar depressive disorder	44 (41.1%)	
- Dysthymia	1 (1.00%)	
- Panic disorder (with/without agoraphobia)	18 (16.8%)	
Received treatment (last episode)*		
- Cognitive behavioural therapy	93 (85.3%)	
- Interpersonal therapy	17 (15.6%)	
- Pharmacotherapy	59 (54.1%)	
- Other kind of therapy (day treatment, EMDR**, CBASP***)	31 (28.4%)	
History of psychiatric treatment	75 (69.4%)	
Number of previous treatment episodes		
- 0	25 (22.9%)	
- 1	28 (25.7%)	
- 2	14 (12.8%)	
- ≥ 3	26 (23.9%)	
- Unknown	8 (7.3%)	
Experience with self-help	21 (19.3%)	
Experience with e-health	6 (5.5%)	
Perceived risk of relapse (percentage)		44.5 (23.1)

*Treatments may be received simultaneously

** EMDR = Eye movement desensitisation reprocessing

*** CBASP = Cognitive Behavioural Analysis System of Psychotherapy

### Estimation results

**Fig 4:**
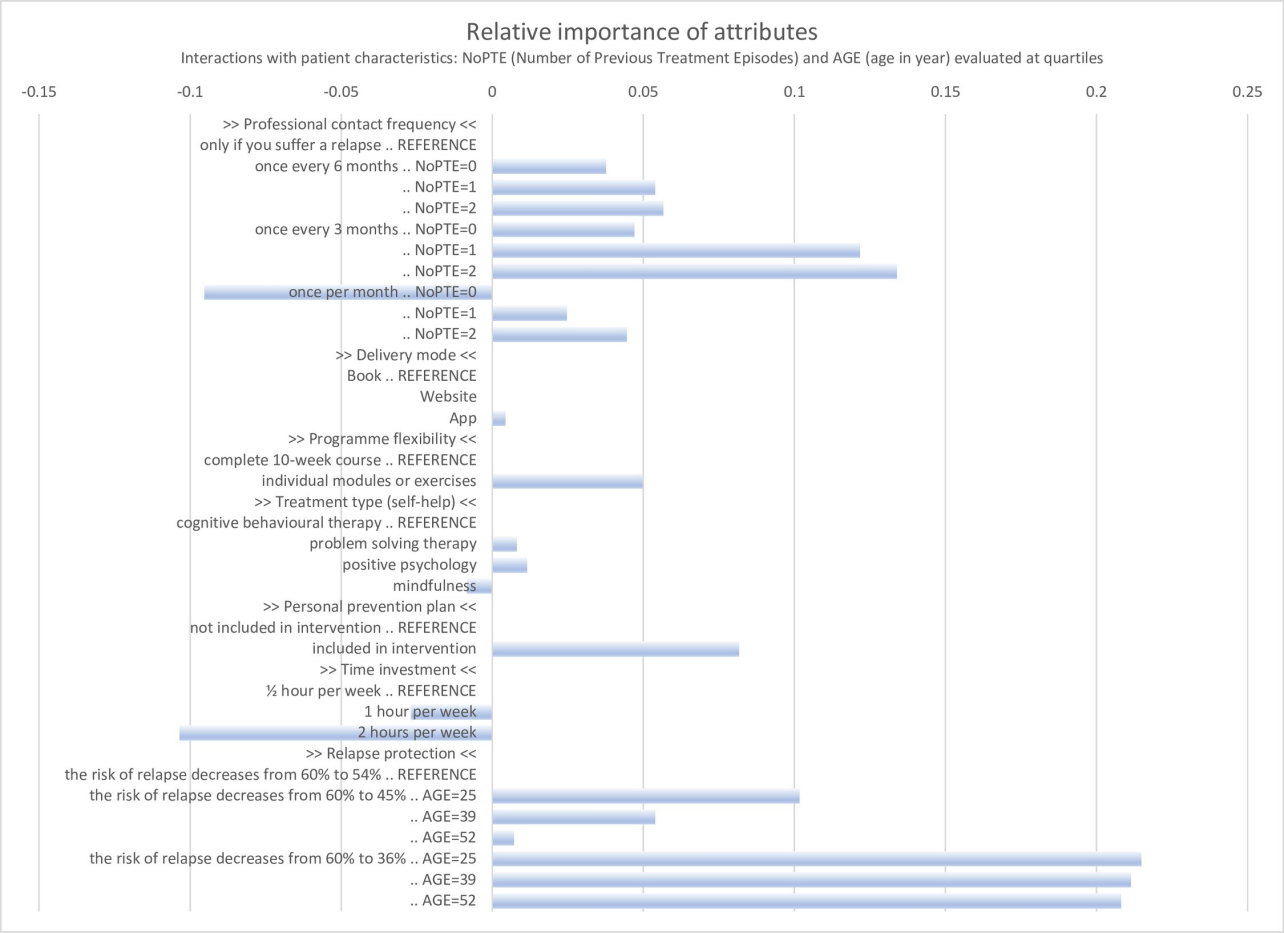
Relative importance of attributes from predicted probability analysis (N = 109). Predicted probabilities are derived from the Mixed Logit model (model 3 in Table 4). For each choice set of each respondent a predicted probability was obtained for each attribute level by changing within a random selected alternative within the choice set the attribute into the successive attribute levels, and averaging the estimated probability of the manipulated alternatives obtained by using the ‘mixlpred’ command over all choice sets and respondents. The attribute level with the lowest predicted probability was selected as the reference category, and for the remaining levels the difference with the average predicted probability of that specific level is depicted, thus showing positive differences in predicted probabilities only.

The results of the DCE based on parameter estimates of the conditional logit models are displayed in Table 4 (models 1 and 2) and the results of the final mixed logit model are displayed in Table 4 (model 3) and Fig 4. Table 4 starts with showing the preference of non-treatment over treatment (the ASC) which is not explained by the attributes, followed by the seven attributes of the DCE. The parameters of the conditional logit model show preferences for lower time investment and higher effectiveness (decreased risk of relapse), which could be expected from an economic perspective. This means that high effectiveness and low/intermediate time investment (<2 hours a week) increased the likelihood of a participant choosing a treatment programme. Furthermore, in the conditional logit model, there were significant preferences for: 1) a contact frequency of once every three months versus only if a relapse occurs; 2) individual modules and exercises versus a complete 10-week programme; and 3) the inclusion of a personal prevention plan versus no prevention plan.

In Models 2 and 3, heterogeneity in preferences was investigated by adding interaction terms to the model using the clinical and socio-demographic characteristics of participants. We discuss the results of the final model (Model 3), because the mixed logit model takes heterogeneity in preferences into account and this model had the best fit.

**Table 4:** Parameter estimates of the conditional logit model with attributes only (model 1), the conditional logit model with attributes and attribute by patient characteristic interaction (model 2) and the mixed logit model with attributes and attribute by patient characteristic interactions (model 3).

	MODEL 1		MODEL 2		MODEL 3			
	Conditional logit		Conditional logit + interactions^2)^		Mixed logit + interactions^3)^			
	est	s.e.	est	s.e.	mean	s.e.	std.dev.	s.e.
Non-treatment versus treatment (ASC)	0.578***	0.133	0.854***	0.176	-0.042	0.486	2.984***	0.244
* Interacted with “had CBT before”			-0.269*	0.129	-0.014	0.472		
* Interacted with “had Internet treatment before”			-0.765**	0.243	-1.639***	0.421		
* Interacted with BAI^1)^			-0.222***	0.053	-0.258	0.137		
Professional contact frequency								
only if you suffer a relapse	reference category							
once every 6 months	0.179	0.095	0.192*	0.097	0.248*	0.121	0.315*	0.150
once every 3 months	0.351***	0.096	0.365***	0.099	0.550***	0.121	0.086	0.164
once per month	0.075	0.096	0.079	0.099	0.123	0.131	0.561**	0.162
* Interacted with NoPTE^1)^: every 6 months			-0.066	0.082	0.105	0.127		
* Interacted with NoPTE ^1)^: every 3 months			0.193**	0.074	0.481***	0.128		
* Interacted with NoPTE ^1)^: every month			0.373***	0.082	0.777***	0.157		
Delivery mode								
Book	reference category							
Website	-0.005	0.086	-0.007	0.089	0.006	0.111	0.312*	0.130
App	0.028	0.074	0.022	0.076	0.027	0.099	0.451**	0.159
Programme flexibility								
complete 10-week course	reference category							
individual modules or exercises	0.183***	0.058	0.199**	0.059	0.260**	0.092	0.724***	0.096
Treatment type (self-help)								
cognitive behavioural therapy	reference category							
problem solving therapy	0.042	0.096	0.060	0.099	0.047	0.121	0.258	0.151
positive psychology	0.072	0.092	0.102	0.094	0.053	0.121	0.451*	0.175
mindfulness	0.034	0.092	0.035	0.095	0.000	0.147	1.125***	0.156
Personal prevention plan								
not included in intervention	reference category							
included in intervention	0.314***	0.060	0.270***	0.062	0.371***	0.079	0.340***	0.076
Time investment								
½ hour per week	reference category							
1 hour per week	-0.091	0.085	-0.074	0.088	-0.118*	^4)^	0.065	
					-2.271*	0.878	0.516	0.404
2 hours per week	-0.312***	0.098	-0.323**	0.101	-0.589*	^4)^	1.326**	
					-1.431*	0.584	1.343**	0.392
Relapse protection								
the risk of relapse decreases from 60% to 54%	reference category							
the risk of relapse decreases from 60% to 45%	0.158*	0.080	0.168*	0.082	0.271***	^4)^	0.159	
					-1.424***	0.390	0.087	0.416
the risk of relapse decreases from 60% to 36%	0.551***	0.105	0.570***	0.109	2.152*	^4)^	11.880***	
					-0.958*	0.405	1.857***	0.351
* Interacted with age^1)^: 60% to 45%			-0.149*	0.059	-0.245**	0.085		
* Interacted with age^1)^: 60% to 36%			-0.148*	0.063	-0.020	0.107		
*Number of patients*	*109*		*105*		*105*			
*Number of observations*	*6447*		*6222*		*6222*			
*Log-likelihood*	*-2299*.*9*		*-2177*.*4*		*-1761*.*2*			
*df*	*15*		*23*		*41*			
*AIC*	*4629*.*8*		*4400*.*8*		*3604*.*4*			
*BIC*	*4731*.*3*		*4555*.*8*		*3890*.*6*			

est = parameter estimate, s.e. = standard error, ASP = alternative specific constant (utility of non-treatment), BAI = Beck Anxiety Inventory, IDS = Inventory of Depressive Symptomatology, NoPTE = Number of previous treatment episodes, df = degrees of freedom, AIC = Akaike Information Criterion, BIC = Bayesian Information Criterion

^1)^ continuous variables were standardized.

^2)^ the model is a result of backward-stepwise method (significance level of removal = 0.05) of estimating the conditional model added all highly significant (i.e. p<0.001) single interactions, being the interaction terms of the ASP with age, age at onset, BAI, IDS, ASI, having received CBT at last episode, having experience with e-health, the interaction terms of professional contact frequency with BAI and with IDS, of treatment type with BAI and with IDS and of Relapse protection with age.

^3)^ the same interaction terms were added to the mixed logit model; in the mixed logit model distributions were assumed normal, however for the attributes (minus) time investment and relapse prevention were lognormal;

^4)^ for reasons of comparison the originally estimated parameters of the lognormal distributions were transformed to display the mean and standard deviation of the lognormal distribution, while the originally estimated parameters and standard errors of the lognormal distribution are displayed at the line below.

***: p-value<0.001;

**: p-value<0.01;

*: p-value<0.05

Model 3 shows that the ASC (preference of non-treatment over treatment), and all attributes exhibit significant standard deviations for one or more levels, which means that there was significant heterogeneity in preferences. Examining the preferences for treatment overall (the ASC) shows that having internet treatment before significantly increased the likelihood of choosing a treatment. With respect to the frequency of professional contact, there was high heterogeneity for the level ‘every month’ which was significantly and positively related to a higher number of previous treatment episodes, which means that patients who had been in treatment multiple times were more likely to choose for a treatment programme with frequent contact with a professional. Preference for a high effectiveness was significantly related to younger age. Preferences were generally low for treatment type, although we observed high heterogeneity for the level ‘mindfulness’. Predicted probability analysis using model 3 in Table 4 indicated that the average probability of preferring the base treatment over non-treatment was 0.517%. Depending on patient characteristics, this probability varied from 0.484 for patients without previous experience regarding internet treatment and a relatively low anxiety severity score (lowest quartile BAI) to 0.702 for patients with previous experience and a relatively high anxiety severity score (highest quartile BAI). Adding a personal prevention plan to the base treatment increased the average probability of preferring the treatment over non-treatment to 0.559, being an 8.2% increase. Fig 4 displays this increase for this attribute and the increase or decrease for other attributes, sometimes interacted with patient characteristics and thus displaying their relative importance. Note that the biggest impact on preferences comes from higher effectiveness (decreased risk of relapse), especially for younger patients. With regard to professional contact frequency, having the second biggest impact, we observe that patients who had previous treatment episodes before (NoPTE = 1 or 2) preferred a more frequent professional contact (once every 3 month or once every month) more than patients who never were treated before (NoPTE = 0), while we also observe a general preference for “once every 3 months”. Fig 4 also shows that the impact of the attributes delivery mode and treatment type are indifferent.

Regarding the issue of the poolability of the two data sets coming from different designs, the Swait and Louviere (SL) test rejected the assumption of equal parameters: already in the first step the SL test showed that differences in parameters could not be resolved by different scale parameters, suggesting that differences between parameters lied elsewhere (see S1 Table). Additional analysis (see S1 Table) showed that adding a single design-by-attribute parameter concerning the ‘personal prevention plan’ resulted in a model that was—in terms of likelihood ratio test—statistically as good as the model containing all design-by-attribute model). Since the parameter estimates based on models that incorporate that specific single design-by-attribute interaction term and the derived figure displaying the relative importance of the attributes are quite similar and lead to the same conclusions (see S2 Table and S1 Fig), we decided to present the results based on the combined data, without the single design-by-attribute interaction term.

### Quality checks

We checked for straight lining: option A was chosen on average 6.6 times (median = 8, IQR = 6 and maximum 13), option B was chosen on average 6.6 times (median = 7, IQR = 5 and maximum 13) and opt-out was chosen on average 6.6 times (median = 4, IQR = 12 and maximum 20). In total, seven patients chose opt-out in all 20 choice sets, while 31 persons never chose opt-out.

Furthermore, a sensitivity analysis (conditional logit model) excluding patients with a score in the category “severe” or “very severe” on the BAI (anxiety) or IDS (depression) was performed, which yielded similar results (Table 5).

**Table 5:** Parameter estimates of the conditional logit model with attributes only estimated on the complete sample (n = 109, model 1), and the conditional logit model with attributes only estimated on subsample excluding patients with scores severe on BAI and/or IDS (n = 95, model 2).

	Clogit (n = 109)		Clogit (n = 95)	
	est	s.e.	est	s.e.
Non-treatment versus treatment (ASP)	0.578***	0.133	0.649***	0.144
Professional contact frequency				
only if you suffer a relapse	Reference			
once every 6 months	0.179	0.095	0.147	0.102
once every 3 months	0.351***	0.096	0.297**	0.104
once per month	0.075	0.096	-0.018	0.105
Delivery mode				
Book	reference			
Website	-0.005	0.086	0.002	0.093
App	0.028	0.074	0.025	0.080
Programme flexibility				
complete 10-week course	reference			
individual modules or exercises	0.183***	0.058	0.218***	0.063
Treatment type (self-help)				
cognitive behavioural therapy	reference			
problem solving therapy	0.042	0.096	0.058	0.104
positive psychology	0.072	0.092	0.065	0.100
mindfulness	0.034	0.092	0.076	0.099
Personal prevention plan				
not included in intervention	reference			
included in intervention	0.314***	0.060	0.233***	0.065
Time investment				
½ hour per week	Reference			
1 hour per week	-0.091	0.085	-0.099	0.093
2 hours per week	-0.312***	0.098	-0.397***	0.107
Relapse protection				
the risk of relapse decreases from 60% to 54%	reference			
the risk of relapse decreases from 60% to 45%	0.158*	0.080	0.228**	0.086
the risk of relapse decreases from 60% to 36%	0.551***	0.105	0.678***	0.115

est = parameter estimate, s.e. = standard error, ASP = alternative specific constant

***: p-value<0.001;

**: p-value<0.01;

*: p-value<0.05

## Discussion

A high effectiveness of a relapse prevention programme, regular meetings with a mental health professional (preferably once every three months), the use of a personal prevention plan and a limited time investment (maximum of one hour per week) were the most important attributes valued by patients partially or fully remitted from anxiety or depressive disorders when choosing to engage in relapse prevention. Results should be interpreted with caution due to a relatively small sample size and high observed heterogeneity in preferences. Examining heterogeneity revealed that having received internet treatment before increased the likelihood of preference for a relapse prevention programme overall, while younger age and a higher number of previous treatment episodes were related to a preference for a high effectiveness and frequent professional contact respectively.

### Strengths and limitations

A strength of this study is that it is the first study to examine preferences for relapse prevention in depression and anxiety using a DCE. This is highly relevant, as evidence-based interventions for relapse prevention either need to be developed (for anxiety) or are not easily accepted by patients (for depression and anxiety). We used recommended standards for designing, administrating and analysing a DCE [29,33].

The most important limitation of this study is the relatively small sample size. Although most DCEs have more than 200 participants, due to practical limitations this was not realistic in the current study leading to a total of 109 participants from two subsamples with different designs, for which the SL test for poolability was rejected. This means that non-significance of attributes may also be explained by the small sample size, especially because of the high heterogeneity found in our model. Results should therefore be interpreted with caution.

Another limitation of this study may be the attribute selection. Although attribute selection was based on a scoping review, 52 interviews with remitted patients and two focus groups, it may still be possible that we did not include all relevant attributes. Using a ranking list to decide on the inclusion of attributes may have increased reliability of the identification of the most important attributes.

We also note that the experiment is of a hypothetical nature and may not entirely resemble real-life behaviour. Unfortunately, we do not have any information on real-life uptake rates. However, all alternatives presented were realistic options and participants were clearly instructed to answer the questions as if they were being offered these interventions in real life. Another limitation of this study is that approximately half of the eligible patients did not participate, either because we were unable to reach them or because they declined participation for various reasons. It seems likely that these patients were, on average, less interested in relapse prevention. This may have led to an overestimation of the proportion of participants willing to engage in relapse prevention. It is unknown to what extent this may have influenced the results concerning relative importance of the attributes.

### Comparison with previous studies

A recent study investigated preferences of pregnant women for the treatment of perinatal depression and anxiety [30]. The authors found that cost and treatment type had the greatest impact on the women’s choices. Although this finding may seem in contrast with our finding that treatment type was relatively unimportant in the choice for relapse prevention, in the study of Ride and Lancsar [30], medication was the treatment type most prominently (and negatively) related to choice, while medication was not one of the options in our study. It is widely established that patients often have a preference for psychological treatment over medication [31]. We did not include medication as an option in our attributes because medication is only viewed as an option for relapse prevention as a continuation treatment and therefore does not apply to patients who remitted after psychological treatment. In contrast to our findings about the non-significance of treatment type, Johansson and colleagues [58] did find a preference for CBT over psychodynamic therapy (both internet-based) and found that the majority of the depressed patients in this pilot study preferred the latter. However, in their study, treatment type was the only difference between the treatment programmes. It seems plausible that patients state a higher preference for attributes such as effectiveness, and time investment compared to treatment type. Nevertheless, when such attributes are equal, treatment type may indeed play a role in choice behaviour and may even be related to outcome [58]. Notably, non-significance of this attribute in our study may also be related to the small sample size and the high heterogeneity of the data. Another explanation treatment type did not emerge as a significant attribute may be that patients did not fully comprehend the content of this attribute, and therefore may have disregarded this attribute in decision making. A finding that contradicts this argument is that 85% of the participants had received cognitive behavioural therapy, so they should be familiar with at least this kind of therapy. Also, an explanation in plain language was provided for each treatment type (see S3 File) and in the read-out-loud pilot, patients seemed to grasp the basic content of the treatment types.

Although most DCE’s include treatment costs we did not include this as an attribute in our study. In the Netherlands, the government has decided that relapse prevention for anxiety or depressive disorders should be offered in the general practice. Mental health care nurses and psychologists working in the general practice are well equipped to support a patient in a low-intensity guided self-help (relapse prevention) programme, as they are able to see the patient at a low frequency spread out over a long period of time. Primary health care is free of charge in the Netherlands and is excluded from the own risk/deductible.

Ride and Lancsar [30] also found that effectiveness was deemed important, while treatment modality was not, which is consistent with our findings.

Age and treatment history were characteristics significantly related to preferences in our study. Having received internet treatment before was related to increased likelihood of treatment uptake and a higher number of previous treatment episodes increased preference for more frequent contact with a professional. Contrary to our expectations, we did not find a relationship between higher symptom severity and professional contact frequency or willingness to invest more time in the intervention. Although the relationship between symptom severity and preference for treatment plus higher contact frequency was present in univariate analysis, this association disappeared in the multivariate models, indicating treatment history played a larger role than symptom severity. It does make sense that patients who have been in treatment multiple times may be more aware of their risk of relapse and may be more familiar and willing to being in (continued) treatment. The relationship between age and a preference for effectiveness is less evident. In previous studies on patient preferences for mental health interventions using a DCE, disease characteristics [20], level of education [59], past experience with mental health treatment [20], employment status [20], ethnicity [59], perceived level of support [20] and attitude towards mental health treatment [20] were related to stated preferences. Since these were all individual studies, more research is needed to explore the relationship between socio-demographic and clinical characteristics and preference for mental health treatment. Our study stresses the importance of personal contact in mental health, as has also been pointed out by a recent review about the acceptability of e-mental health [32]. If patients are to be encouraged to consider the use of e-mental health or other forms of self-help, personal guidance from a professional and a personal approach seem to be crucial for uptake and acceptability.

### Implications for research and clinical practice

Our study revealed important information about patient preferences when presented different treatment options for relapse prevention. Most importantly, patients place high importance on effectiveness of treatment, regular contact with a professional, a feasible time investment and a personal treatment plan. It seems that less emphasis is placed on treatment type and delivery mode. Although it is too early to conclude that patients with common mental disorders in general do not have a preference for treatment modality, this may indicate that personalising interventions and enhancing informed choice may have a larger impact on the uptake of interventions than offering different treatment modalities.

Informing patients about the content and effectiveness of interventions may increase acceptability in general, but the most efficient way to achieve this should be studied further. For example, Casey and colleagues found that a textual explanation of an e-mental health programme increased the likelihood of acceptance but an education film did not [60]. Other tools that may improve the decision-making process are: 1) presenting information about effectiveness in easily understandable numbers (such as event rates); and 2) using graphic information [61]. Shared decision-making could play a significant role in this process, by informing patients about the pros and cons of treatment alternatives and enabling patients to make an informed choice [62]. In line with previous studies, our study indicated that there is a high heterogeneity in preferences; in fact, heterogeneity was significant on all attributes. This means that differences in preferences should be taken into account when offering treatment options to patients. Although a bit preliminary, based on our results it may be worthwhile to pay special attention to informing younger patients about the effectiveness of treatment options, while offering patients with more residual symptoms a higher frequency of (booster) sessions.

### Conclusions

A DCE is an elegant method for identifying preferences of patients. This study is one of the first DCE’s in the mental health field, yielding interesting results regarding patient preferences for relapse prevention and heterogeneity in preferences. The results of this study can be used to guide the development of a relapse prevention programme for anxiety and depressive disorders.

## Supporting information

S1 Table Estimated parameters of different conditional logit models. (DOCX)

S2 Table Parameter estimates of models that also include the “personal prevention plan” by design interaction term to address the issue of poolability. (DOCX)

S1 Fig Relative importance attributes from the model that includes the “personal prevention plan” by design interaction term to address the issue of poolability. (DOCX)

S1 File Interview guides for focus groups with professionals and patients. (DOCX)

S2 File Rating list of 30 hypothetical treatment attributes of a relapse prevention programme. (DOC)

S3 File Final survey including DCE. (DOCX)

S4 File Final Bayesian efficient design (Ngene syntax). (DOCX)

S5 File Rating list of 30 hypothetical treatment attributes of a relapse prevention programme [in Dutch]. (DOCX)

S6 File Final survey including DCE. (DOC)
