# Supplementary material for: Patient preferences for a guided self-help programme to prevent relapse in anxiety or depression: A discrete choice experiment
Source: PLoS One. 2019 Jul 18;14(7):e0219588. doi: 10.1371/journal.pone.0219588 (PMC6638925; doi:10.1371/journal.pone.0219588)
Supplement: S1 Table — (DOCX) [file pone.0219588.s001.docx]

S1 Table. Estimated parameters of different conditional logit models: using combined data of designs 1 and 2 (Model 1), using data of ‘Design 1’ only, data of ‘Design 2’ only, using combined data with scale parameter (Heterosced.), with a ‘Fully interacted model’ and with a restricted model (Model 1a)

|  | Model 1 | |  | Design 1 | |  | Design 2 | |  | Heterosced. | |  | Fully interacted model | | | |  | Model 1a | | | |
| --- | --- | --- | --- | --- | --- | --- | --- | --- | --- | --- | --- | --- | --- | --- | --- | --- | --- | --- | --- | --- | --- |
|  |  |  |  |  |  |  |  |  |  |  |  |  | Main | | Interaction | |  | Main | | Interaction | |
|  |  |  |  |  |  |  |  |  |  |  |  |  | effects | | effects | |  | effects | | effects | |
|  | b | s.e. |  | b | s.e. |  | b | s.e. |  | b | s.e. |  | b | s.e. | b | s.e. |  | b | s.e. | b | s.e. |
| Non-treatment versus treatment (ASC) | 0.58 | 0.13 |  | 0.09 | 0.35 |  | 0.80 | 0.21 |  | 0.55 | 0.15 |  | 0.80 | 0.21 | -0.71 | 0.41 |  | 0.61 | 0.13 |  |  |
| Professional contact frequency |  |  |  |  |  |  |  |  |  |  |  |  |  |  |  |  |  |  |  |  |  |
| only if you suffer a relapse |  |  |  |  |  |  |  |  |  |  |  |  |  |  |  |  |  |  |  |  |  |
| once every 6 months | 0.18 | 0.09 |  | 0.07 | 0.19 |  | 0.19 | 0.16 |  | 0.17 | 0.09 |  | 0.19 | 0.16 | -0.12 | 0.25 |  | 0.16 | 0.10 |  |  |
| once every 3 months | 0.35 | 0.10 |  | 0.31 | 0.20 |  | 0.32 | 0.17 |  | 0.34 | 0.10 |  | 0.32 | 0.17 | -0.02 | 0.26 |  | 0.33 | 0.10 |  |  |
| once per month | 0.07 | 0.10 |  | 0.15 | 0.23 |  | 0.06 | 0.15 |  | 0.08 | 0.09 |  | 0.06 | 0.15 | 0.09 | 0.27 |  | 0.08 | 0.10 |  |  |
| Delivery mode |  |  |  |  |  |  |  |  |  |  |  |  |  |  |  |  |  |  |  |  |  |
| Book |  |  |  |  |  |  |  |  |  |  |  |  |  |  |  |  |  |  |  |  |  |
| Website | 0.00 | 0.09 |  | 0.02 | 0.22 |  | 0.01 | 0.15 |  | 0.00 | 0.08 |  | 0.01 | 0.15 | 0.01 | 0.26 |  | 0.03 | 0.09 |  |  |
| App | 0.03 | 0.07 |  | -0.01 | 0.21 |  | 0.02 | 0.09 |  | 0.03 | 0.07 |  | 0.02 | 0.09 | -0.03 | 0.23 |  | 0.03 | 0.07 |  |  |
| Programme flexibility |  |  |  |  |  |  |  |  |  |  |  |  |  |  |  |  |  |  |  |  |  |
| complete 10-week course |  |  |  |  |  |  |  |  |  |  |  |  |  |  |  |  |  |  |  |  |  |
| individual modules or exercises | 0.18 | 0.06 |  | 0.23 | 0.12 |  | 0.16 | 0.09 |  | 0.18 | 0.06 |  | 0.16 | 0.09 | 0.07 | 0.15 |  | 0.18 | 0.06 |  |  |
| Treatment type (self-help) |  |  |  |  |  |  |  |  |  |  |  |  |  |  |  |  |  |  |  |  |  |
| cognitive behavioural therapy |  |  |  |  |  |  |  |  |  |  |  |  |  |  |  |  |  |  |  |  |  |
| problem solving therapy | 0.04 | 0.10 |  | -0.05 | 0.18 |  | 0.08 | 0.14 |  | 0.04 | 0.09 |  | 0.08 | 0.14 | -0.13 | 0.22 |  | 0.04 | 0.10 |  |  |
| positive psychology | 0.07 | 0.09 |  | -0.18 | 0.17 |  | 0.20 | 0.13 |  | 0.06 | 0.09 |  | 0.20 | 0.13 | -0.38 | 0.21 |  | 0.09 | 0.09 |  |  |
| Mindfulness | 0.03 | 0.09 |  | -0.14 | 0.23 |  | 0.12 | 0.13 |  | 0.02 | 0.10 |  | 0.12 | 0.13 | -0.26 | 0.26 |  | 0.02 | 0.09 |  |  |
| Personal prevention plan |  |  |  |  |  |  |  |  |  |  |  |  |  |  |  |  |  |  |  |  |  |
| not included in intervention |  |  |  |  |  |  |  |  |  |  |  |  |  |  |  |  |  |  |  |  |  |
| included in intervention | 0.31 | 0.06 |  | 0.57 | 0.13 |  | 0.13 | 0.09 |  | 0.32 | 0.06 |  | 0.13 | 0.09 | 0.44 | 0.16 |  | 0.14 | 0.07 | 0.52 | 0.10 |
| Time investment |  |  |  |  |  |  |  |  |  |  |  |  |  |  |  |  |  |  |  |  |  |
| ½ hour per week |  |  |  |  |  |  |  |  |  |  |  |  |  |  |  |  |  |  |  |  |  |
| 1 hour per week | -0.09 | 0.09 |  | -0.28 | 0.16 |  | -0.01 | 0.14 |  | -0.10 | 0.08 |  | -0.01 | 0.14 | -0.27 | 0.22 |  | -0.11 | 0.09 |  |  |
| 2 hours per week | -0.31 | 0.10 |  | -0.36 | 0.27 |  | -0.32 | 0.15 |  | -0.31 | 0.09 |  | -0.32 | 0.15 | -0.03 | 0.31 |  | -0.35 | 0.10 |  |  |
| Relapse protection |  |  |  |  |  |  |  |  |  |  |  |  |  |  |  |  |  |  |  |  |  |
| risk of relapse decreases from 60% to 54% |  |  |  |  |  |  |  |  |  |  |  |  |  |  |  |  |  |  |  |  |  |
| risk of relapse decreases from 60% to 45% | 0.16 | 0.08 |  | -0.02 | 0.21 |  | 0.32 | 0.11 |  | 0.15 | 0.08 |  | 0.32 | 0.11 | -0.34 | 0.24 |  | 0.23 | 0.08 |  |  |
| risk of relapse decreases from 60% to 36% | 0.55 | 0.10 |  | 0.41 | 0.26 |  | 0.76 | 0.20 |  | 0.53 | 0.11 |  | 0.76 | 0.20 | -0.35 | 0.33 |  | 0.66 | 0.11 |  |  |
| Scale parameter |  |  |  |  |  |  |  |  |  | 0.11 | 0.32 |  |  |  |  |  |  |  |  |  |  |
| Log-likelihood model | -2299.9 | |  | -653.0 | |  | -1625.7 | |  | -2299.8 | |  | -2278.7 | | | |  | -2287.0 | | | |

To assess the assumption of poolability of the two data sets for using the conditional logit (CL) model (Model 1) based on the combined data, we estimated CL models for the two data sets separately (Design 1 and Design 2) and a heteroscedastic CL model with a scale parameter for the combined set (Heterosced.). The Swait-Louviere test rejected the null hypothesis that differences in parameters could be resolved using different scale parameters (chi2(14)=-2*(-2299.8 – (-653.0 + -1625.7)) = 42.4, *p*-value = 0.0002). The CL model estimated on the combined data containing all design-by-attribute interaction terms (Fully interacted model) evaluates differences for all attributes. The simpler CL model containing only the design-by-personal-prevention-plan interaction term (Model 1a) could not be discriminated from the fully interacted model, given the data (likelihood ratio test statistic: chi2(14) = 16.74, p-value=0.2701.
