# Supplementary material for: Patient preferences for a guided self-help programme to prevent relapse in anxiety or depression: A discrete choice experiment
Source: PLoS One. 2019 Jul 18;14(7):e0219588. doi: 10.1371/journal.pone.0219588 (PMC6638925; doi:10.1371/journal.pone.0219588)
Supplement: S2 Table — (DOCX) [file pone.0219588.s002.docx]

S2 Table. Parameter estimates of models that also include the “personal prevention plan” by design interaction term to address the issue of poolability:. the conditional logit model with attributes only (MODEL 1a), the conditional logit model with attributes and attribute by patient characteristic interaction (MODEL 2a) and the mixed logit model with attributes and attribute by patient characteristic interactions (MODEL 3a)

|  | **MODEL 1a** | |  | **MODEL 2a** | |  | **MODEL 3a** | | | |
| --- | --- | --- | --- | --- | --- | --- | --- | --- | --- | --- |
|  | **Conditional logit** | |  | **Conditional logit + interactions**^2)^ | |  | **Mixed logit + interactions**^3)^ | | | |
|  | **Est** | **s.e.** |  | **est** | **s.e.** |  | **mean** | **s.e.** | **std.dev.** | **s.e.** |
| Non-treatment versus treatment (ASC) | 0.610*** | 0.134 |  | 0.642*** | 0.138 |  | 0.520* | 0.248 | 3.062*** | 0.251 |
| * Interacted with “had Internet treatment before” |  |  |  | -0.743** | 0.243 |  | -2.060*** | 0.572 |  |  |
| * Interacted with BAI^1)^ |  |  |  | -0.229*** | 0.052 |  | -1.052*** | 0.193 |  |  |
| Professional contact frequency |  | | | | | | | | | |
| only if you suffer a relapse | reference category | | | | | | | | | |
| once every 6 months | 0.163 | 0.096 |  | 0.181 | 0.098 |  | 0.325* | 0.130 | 0.030 | 0.297 |
| once every 3 months | 0.328** | 0.097 |  | 0.348*** | 0.099 |  | 0.708*** | 0.146 | 0.621*** | 0.153 |
| once per month | 0.082 | 0.097 |  | 0.082 | 0.100 |  | 0.144 | 0.158 | 1.060*** | 0.151 |
| * Interacted with NoPTE^1)^: every 6 months |  |  |  | -0.068 | 0.082 |  | 0.136 | 0.134 |  |  |
| * Interacted with NoPTE ^1)^: every 3 months |  |  |  | 0.193** | 0.074 |  | 0.652*** | 0.160 |  |  |
| * Interacted with NoPTE ^1)^: every month |  |  |  | 0.372*** | 0.082 |  | 0.940*** | 0.185 |  |  |
| Delivery mode |  | | | | | | | | | |
| Book | reference category | | | | | | | | | |
| Website | 0.026 | 0.086 |  | 0.014 | 0.089 |  | -0.006 | 0.132 | 0.452** | 0.159 |
| App | 0.034 | 0.074 |  | 0.025 | 0.076 |  | 0.057 | 0.114 | 0.641*** | 0.124 |
| Programme flexibility |  | |  | | |  |  |  |  |  |
| complete 10-week course | reference category | | | | | | | | | |
| individual modules or exercises | 0.182** | 0.058 |  | 0.197** | 0.060 |  | 0.408*** | 0.110 | 0.854*** | 0.112 |
| Treatment type (self-help) |  | |  | | |  |  |  |  |  |
| cognitive behavioural therapy | reference category | | | | | | | | | |
| problem solving therapy | 0.041 | 0.097 |  | 0.058 | 0.099 |  | 0.086 | 0.137 | 0.385* | 0.157 |
| positive psychology | 0.090 | 0.092 |  | 0.112 | 0.095 |  | 0.119 | 0.144 | 0.769*** | 0.129 |
| mindfulness | 0.018 | 0.093 |  | 0.026 | 0.095 |  | -0.056 | 0.168 | 1.317*** | 0.167 |
| Personal prevention plan |  | |  | | |  |  |  |  |  |
| not included in intervention | reference category | | | | | | | | | |
| included in intervention | 0.136 | 0.070 |  | 0.256*** | 0.062 |  | 0.464*** | 0.107 | 0.592*** | 0.105 |
| * old design | 0.520*** | 0.102 |  | 0.335** | 0.107 |  | 0.416* | 0.202 |  |  |
| Time investment |  | | | | | | | | | |
| ½ hour per week | reference category | | | | | | | | | |
| 1 hour per week | -0.113 | 0.086 |  | -0.088 | 0.088 |  | -0.105 | ^4)^ | 0.240 |  |
|  |  |  |  |  |  |  | -3.174 | 2.400 | 1.353 | 1.157 |
| 2 hours per week | -0.353*** | 0.099 |  | -0.348** | 0.102 |  | -0.737* | ^4)^ | 2.584** |  |
|  |  |  |  |  |  |  | -1.599* | 0.721 | 1.609** | 0.482 |
| Relapse protection |  | |  | | |  |  |  |  |  |
| the risk of relapse decreases from 60% to 54% | reference category | | | | | | | | | |
| the risk of relapse decreases from 60% to 45% | 0.228** | 0.081 |  | 0.210* | 0.083 |  | 0.366* | ^4)^ | 0.535 |  |
|  |  |  |  |  |  |  | -1.575* | 0.660 | 1.069* | 0.494 |
| the risk of relapse decreases from 60% to 36% | 0.662*** | 0.108 |  | 0.637*** | 0.111 |  | 1.385 | ^4)^ | 2.666*** |  |
|  |  |  |  |  |  |  | -0.448 | 0.298 | 1.244*** | 0.206 |
| * Interacted with age^1)^: 60% to 45% |  |  |  | -0.164** | 0.058 |  | -0.268* | 0.109 |  |  |
| * Interacted with age^1)^: 60% to 36% |  |  |  | -0.165** | 0.063 |  | -0.138 | 0.127 |  |  |
| *Number of observations* | *6447* |  |  | *6222* |  |  | *6222* |  |  |  |
| *Number of observations* | *6447* |  |  | *6222* |  |  | *6222* |  |  |  |
| *Log-likelihood* | *-2287.0* |  |  | *-2174.7* |  |  | *-1622.14* |  |  |  |
| *df* | *16* |  |  | *23* |  |  | *38* |  |  |  |
| *AIC* | *4605.9* |  |  | *4395.5* |  |  | *3320.3* |  |  |  |
| *BIC* | *4714.3* |  |  | *4550.4* |  |  | *3576.3* |  |  |  |

est = parameter estimate, s.e.=standard error, ASC=alternative specific constant (utility of non-treatment), BAI = Beck Anxiety Inventory, IDS = Inventory of Depressive Symptomatology, NoPTE = Number of previous treatment episodes, df = degrees of freedom, AIC = Akaike Information Criterion, BIC = Bayesian Information Criterion ^1)^ continuous variables were standardized. ^2)^ the model is a result of backward-stepwise method (significance level of removal = 0.05) of estimating the conditional model added all highly significant (i.e. p<0.001) single interactions, being the interaction terms of the ASP with age, age at onset, BAI, IDS, ASI, having received CBT at last episode, having experience with e-health, the interaction terms of professional contact frequency with BAI and with IDS, of treatment type with BAI and with IDS and of Relapse protection with age. ^3)^ the same interaction terms were added to the mixed logit model; in the mixed logit model distributions were assumed normal, however for the attributes (minus) time investment and relapse prevention were lognormal; ^4)^ for reasons of comparison the originally estimated parameters of the lognormal distributions were transformed to display the mean and standard deviation of the lognormal distribution, while the originally estimated parameters and standard errors of the lognormal distribution are displayed at the line below.

***: p-value<0.001; **: p-value<0.01; *: p-value<0.05
