# Supplementary material for: Patient preferences for a guided self-help programme to prevent relapse in anxiety or depression: A discrete choice experiment
Source: PLoS One. 2019 Jul 18;14(7):e0219588. doi: 10.1371/journal.pone.0219588 (PMC6638925; doi:10.1371/journal.pone.0219588)
Supplement: S1 Fig — (DOCX) [file pone.0219588.s003.docx]

S1 Fig. Relative importance attributes from the model (MODEL 3a) that includes the “personal prevention plan” by design interaction term to address the issue of poolability


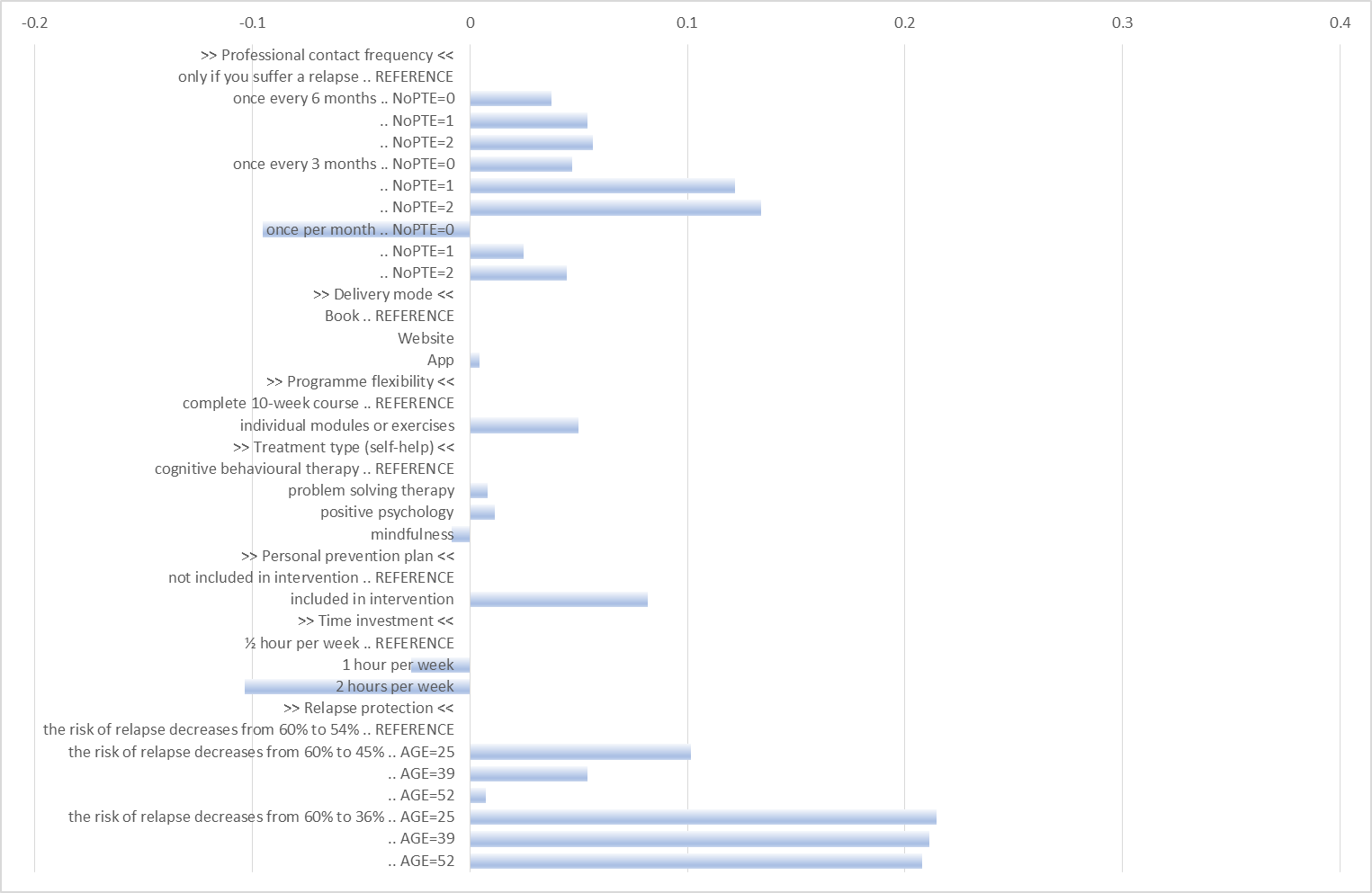


Interaction with patient characteristics: NoPTE = Number of Previous Treatment Episodes (First Quartile = 0; Median = 1; Third Quartile=2); Age (First Quartile=25, Median=39; Third Quartile=52)
