# Supplementary material for: Patient preferences for a guided self-help programme to prevent relapse in anxiety or depression: A discrete choice experiment
Source: PLoS One. 2019 Jul 18;14(7):e0219588. doi: 10.1371/journal.pone.0219588 (PMC6638925; doi:10.1371/journal.pone.0219588)
Supplement: S1 File — (DOCX) [file pone.0219588.s004.docx]

**Supplement 1. Interview guide for focus groups with professionals and patients**

**Professionals**

1. Introduction and explanation focus group

Main question: What should an individual, guided-self help relapse prevention intervention for anxiety or depression supervised by a professional look like?

2. Group discussion.

Questions:
- What makes it attractive for patients to participate?

- What elements should be included in this intervention?

- What should the guidance of the professional encompass?

Subtopics of interest:
- Self-help through internet, app or book
- Mode of contact with patient (mail, telephone, face-to-face)
- Frequency of contact
- Monitoring of symptoms
- Medication monitoring

3. Professionals are invited to write the important elements of a guided-self help relapse prevention intervention on a post-it.
- 10 post-its per person

- focus group leaders collect post-its and regroup themes on an A0 paper

4. Professionals all receive 5 stickers. They are invited to place a sticker on the five elements they deem most important.

5. Discussion about the elements.

6. Closing.

**Patients**

1. Introduction and explanation focus group

Main question: What should an individual, guided-self help relapse prevention intervention for anxiety or depression supervised by a professional look like?

2. Group discussion

Subtopics of interest:
- Would you be willing to use a website to support you in relapse prevention? Why yes / not? How about a mobile application or a self-help book?
- How should such a website look like? (e.g. modular program or a platform?)
- What kind of professional should provide guidance? (e.g. professional in specialized mental health care, mental health nurse working in primary care)
- Content of intervention (e.g. psycho-education, exercises, information about medication, focused on the anxiety or depressive symptoms / focused on what was previously learned in the therapy / focused on personal growth)
- Mode of contact with the professional (mail, telephone, face-to-face)
- Frequency of contact
- Monitoring of symptoms
- How/when to contact the professional
- Medication monitoring

3. Patients are invited to write the important elements of a guided-self help relapse prevention intervention on a post-it.
- 10 post-its per person

- focus group leaders collect post-its and regroup themes on an A0 paper

4. Professionals all receive 5 stickers. They are invited to place a sticker on the five elements they deem most important.

5. Discussion about the elements.

6. Closing.
