# Supplementary material for: Patient preferences for a guided self-help programme to prevent relapse in anxiety or depression: A discrete choice experiment
Source: PLoS One. 2019 Jul 18;14(7):e0219588. doi: 10.1371/journal.pone.0219588 (PMC6638925; doi:10.1371/journal.pone.0219588)
Supplement: S2 File — (DOC) [file pone.0219588.s005.doc]

**Supplement 2. Rating list of 30 hypothetical treatment attributes of a relapse prevention program**

*The following questions concern your preferences for a maintenance treatment programme after you have finished therapy at GGZ inGeest. This treatment programme is currently in development and will be made available to patients in the future. The maintenance treatment programme could comprise various components.*

**Please specify for each of the following components whether you think it should be included in a maintenance treatment programme after finishing therapy at GGZ inGeest.**

| **After I finished therapy at GGZ inGeest, it is important for me …** | Completely disagree | Disagree | Neither agree nor disagree | Agree | Completely agree |
| --- | --- | --- | --- | --- | --- |
| ... to have a dedicated therapist who I can contact | 1 | 2 | 3 | 4 | 5 |
| ... to decide for myself how many sessions I need with the therapist in a year | 1 | 2 | 3 | 4 | 5 |
| ... to be able to contact a therapist by email | 1 | 2 | 3 | 4 | 5 |
| ... that my new therapist is aware of what I have learned during my previous therapy | 1 | 2 | 3 | 4 | 5 |

| **After I have finished therapy at GGZ inGeest, I would like to have access to …** | Completely disagree | Disagree | Neither agree nor disagree | Agree | Completely agree |
| --- | --- | --- | --- | --- | --- |
| ... a smartphone app with information, exercises and tips to prevent a relapse | 1 | 2 | 3 | 4 | 5 |
| ... a self-help book with information, exercises and tips to prevent a relapse | 1 | 2 | 3 | 4 | 5 |
| ... a website with information, exercises and tips to prevent a relapse | 1 | 2 | 3 | 4 | 5 |

| **My choice to follow a maintenance treatment programme depends on ...** | Completely disagree | Disagree | Neither agree nor disagree | Agree | Completely agree |
| --- | --- | --- | --- | --- | --- |
| ... the amount of time I have to spend on the programme | 1 | 2 | 3 | 4 | 5 |
| ... how much the programme reduces my risk of a relapse | 1 | 2 | 3 | 4 | 5 |
| ... whether my insurance covers the programme | 1 | 2 | 3 | 4 | 5 |

| **I will be more likely to use a website to get support if …** | Completely disagree | Disagree | Neither agree nor disagree | Agree | Completely agree |
| --- | --- | --- | --- | --- | --- |
| ... it includes exercises I can do to help me practice what I learned during therapy | 1 | 2 | 3 | 4 | 5 |
| ... my therapist helps me to use the website | 1 | 2 | 3 | 4 | 5 |
| ... my therapist provides me with feedback on the website exercises I complete | 1 | 2 | 3 | 4 | 5 |
| … I can use it to prepare a personal plan to prevent relapse (relapse prevention plan) | 1 | 2 | 3 | 4 | 5 |
| ... the exercises help me to learn how to solve my own problems | 1 | 2 | 3 | 4 | 5 |
| ... the exercises help me to change negative thoughts and behaviour | 1 | 2 | 3 | 4 | 5 |
| ... the exercises help me to learn how to live in the here and now | 1 | 2 | 3 | 4 | 5 |
| ... the exercises help me to give my life direction | 1 | 2 | 3 | 4 | 5 |
| ... it offers a complete course that comprises a fixed number of lessons | 1 | 2 | 3 | 4 | 5 |
| ... it offers various exercises and courses that I can choose from as I see fit | 1 | 2 | 3 | 4 | 5 |
| ... I get reminded to do the exercises by text message or email | 1 | 2 | 3 | 4 | 5 |
| ... I can chat with other current and former patients | 1 | 2 | 3 | 4 | 5 |
| ... I can contact a psychiatrist through the website | 1 | 2 | 3 | 4 | 5 |
| ... I can use it on my tablet | 1 | 2 | 3 | 4 | 5 |
| ... it is visually attractive | 1 | 2 | 3 | 4 | 5 |
| ... it is easy to use | 1 | 2 | 3 | 4 | 5 |
| ... it is clearly structured | 1 | 2 | 3 | 4 | 5 |
| ... I can download the information and exercises | 1 | 2 | 3 | 4 | 5 |
| ... I can share information on it with family/friends/caregivers | 1 | 2 | 3 | 4 | 5 |
| ... I can use it to monitor if my condition is worsening | 1 | 2 | 3 | 4 | 5 |

| **My choice to follow a maintenance treatment programme also depends on ... (Please specify)** |
| --- |
|  |
|  |
|  |
|  |
|  |
