# Supplementary material for: Patient preferences for a guided self-help programme to prevent relapse in anxiety or depression: A discrete choice experiment
Source: PLoS One. 2019 Jul 18;14(7):e0219588. doi: 10.1371/journal.pone.0219588 (PMC6638925; doi:10.1371/journal.pone.0219588)
Supplement: S3 File — (DOCX) [file pone.0219588.s006.docx]

**Questionnaire about maintenance treatment after therapy**

Research reveals that people who have suffered from an anxiety disorder or depression have a high risk of relapse. Approximately 1 in every 4 patients who recover suffer a relapse within 2 years and as many as 60% suffer a relapse of the anxiety or depressive disorder within 4 years. Patients who follow maintenance treatment have a decreased likelihood of suffering a relapse. However, no research has yet been conducted into what support patients would prefer to receive after they have finished their therapy at GGZ.

We have created this survey to find out what the preferences and opinions of current and former patients are in regard to maintenance treatment programmes. This questionnaire is intended to provide us with more insight into the support you as a patient (or former patient) would like to receive after completing therapy. To do this, we ask for your opinion about various maintenance treatment options. All information you provide will be treated with the utmost confidentiality and participation in the survey will have no effect on your current therapy programme.

The questionnaire starts with a number of general questions, after which you will be asked to read some specific information about various maintenance treatment options. You are asked to indicate which maintenance treatment option you find the most appealing. Finally, you will be asked to complete four groups of questions that concern your *current* experience of anxiety and/or depression.

It will take approximately 60 minutes to complete the questionnaire. If you have any questions you can ask the research assistant. Thank you very much for participating in this survey!

Dr. A. Muntingh, researcher GGZ inGeest

**Personal details**

1. Date: (day/month/year) ……./…../…………
2. Gender:  Male  Female
3. What is your age? …… years
4. What condition are you or were you being treated for? (You can give more than one answer)

 Anxiety

 Depression

1. What treatment have you received for your condition? (You can give more than one answer)

 cognitive behavioural therapy

 interpersonal therapy

 medication

 Other (please specify) ……………………………………………………………………

1. Have you been treated for this condition before by a psychologist, psychiatrist or GGZ institution?

 yes, …… times

 no

1. Have you ever followed a self-help therapy programme or online therapy for your depression or anxiety? (You can give more than one answer)

 Self-help therapy

 Online therapy

 Neither

1. At what age did you first suffer from depression or anxiety?

|  |
| --- |

Age:

1. What is your highest level of education? (Circle one answer)

1. Primary school, primary education

2. Preparatory vocational education

3. Upper vocational secondary education

4. Senior secondary vocational education and training

5. Senior secondary school

6. Higher professional education or university

7. Other (please specify) ......

1. Do any other close family members of yours (brother/sister/parent/child) suffer from depression or anxiety?

 no, no other members of my family have psychological problems *(continue with question 11)*

 yes, other members of my family also have psychological problems

*Please state the type of disorder (depression or anxiety) and which family member is affected:*

1. (family member) ………………………………………… suffers from (disorder) …………………
2. (family member) ………………………………………… suffers from (disorder) …………………
3. (family member) ………………………………………… suffers from (disorder) …………………
4. (family member) ………………………………………… suffers from (disorder) …………………
5. In your opinion, what is the **risk** of you suffering from a relapse of your depression or anxiety disorder within the coming 4 years? (Place a cross on the line above the appropriate answer)

|--------------------------------------------------------------------------------------------------------------|
0% 10% 20% 30% 40% 50% 60% 70% 80% 90% 100%

**Explanation of the maintenance treatment programmes**

Research reveals that people who have suffered from an anxiety or depressive disorder , and who follow a maintenance treatment programme after they have completed therapy, have a lower risk of relapse. The maintenance treatment programme studied as part of this research involved **personal sessions** with a psychiatric nurse or psychologist in a GP practice, supplemented with the provision of information and exercises using a self-help book or website. We want to know exactly what patients and former patients expect of such a maintenance treatment programme so we can use your input to develop a new programme. Please take your time to read the information below. The research assistant can help you to complete the questionnaire if needed.

If you were offered a maintenance treatment programme after you completed therapy, whereby you would attend personal sessions with a psychiatric nurse or psychologist in your local GP practice and be provided with self-help exercises to do at home, would you participate?

 Yes, I think so

 No, I don’t think so

**Components of the maintenance treatment programme**

**1. Frequency of the sessions with a therapist in the GP practice**

A component of the maintenance treatment programme involves personal sessions (face-to-face) with a therapist (psychologist or psychiatric nurse) who works at your GP practice, so you can discuss with the therapist know how you are doing. The therapist can also provide assistance with any self-help exercises you have been given.

How often would you prefer to meet face-to-face with your therapist?

 Once per month

 Every 3 months

 Every 6 months

 Only if I suffer a relapse

**2. Type of self-help**

Self-help forms an important component of the maintenance treatment programme (in addition to personal sessions with your therapist). The self-help component empowers you to prevent a relapse yourself by learning skills that can help you to deal with the symptoms. There are various types of self-help. The therapist at the GP practice will provide you with support throughout the self-help programme.

- Using a mobile phone **app**. This app provides information and contains exercises that can help you deal with symptoms of anxiety or depression.

*Example exercise:*

- Using a **website**. You will have access to a personal webpage that you log in to with a username and password. The website provides information and contains exercises that can help you deal with symptoms of anxiety or depression.

*Example webpage:*


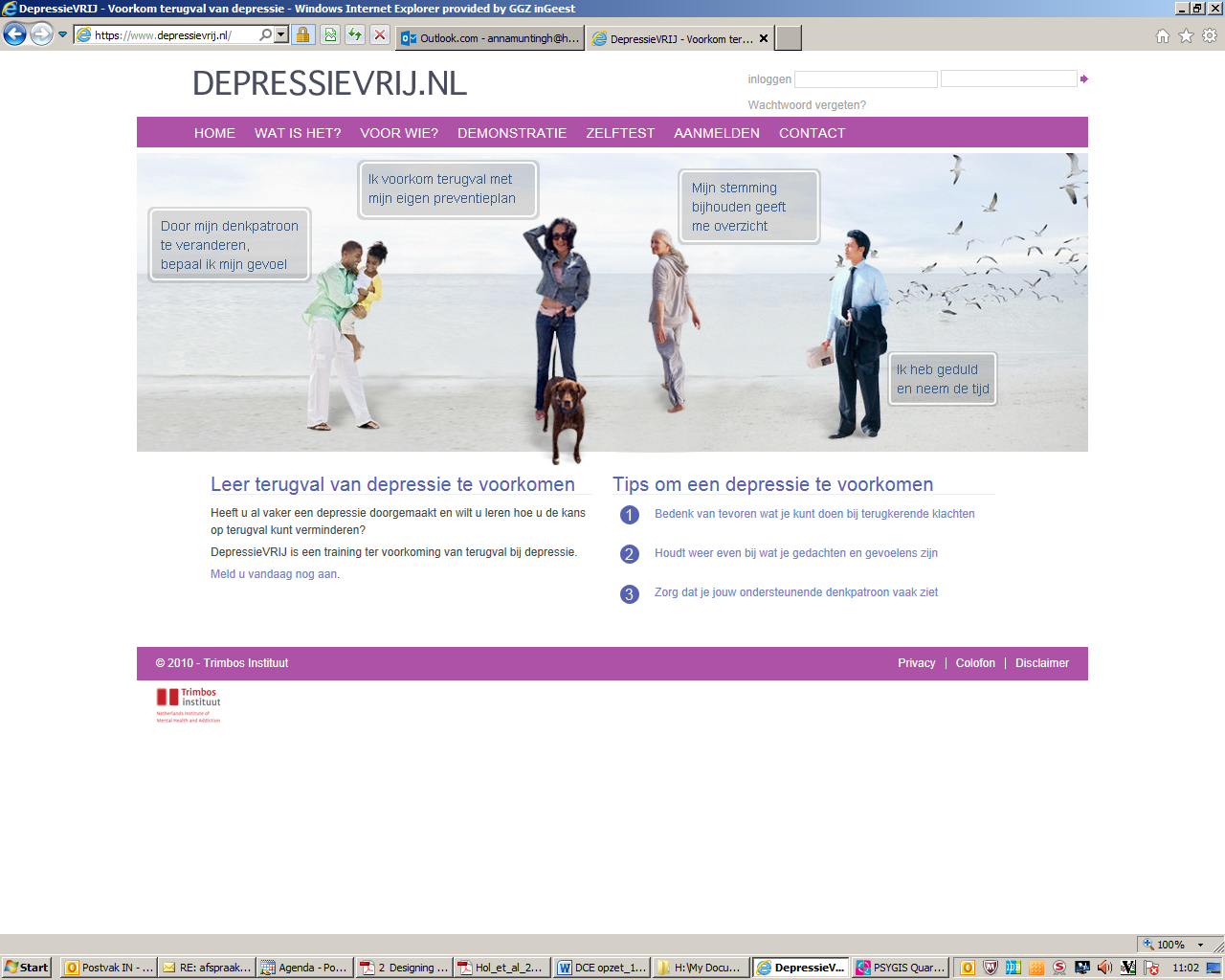


Leer terugval te voorkomen

Heeft u een depressie of angststoornis doorgemaakt en wit u leren hoe u de kans op terugval kunt verminderen? Volg dan deze training ter voorkoming van terugval bij depressie en angst.

Tips om terugval te voorkomen

- Using a **book**. You purchase a self-help book which you can work through step-by-step in your own tempo. The book contains tips and advice for dealing with your symptoms. Your therapist will support you throughout the self-help programme.

*Example of a book*


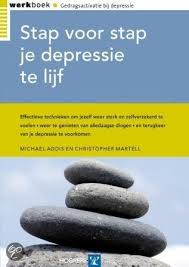


- Using a **self-help book**. You are given a book with information and exercises that can help you deal with symptoms of anxiety or depression.


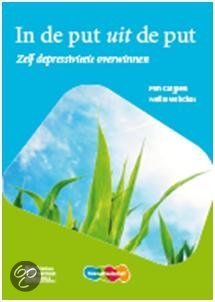
*Examples of self-help books:*

*
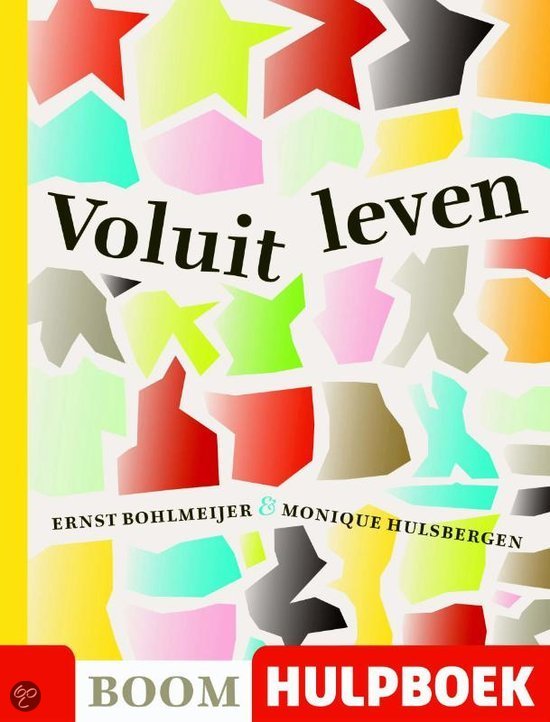
*
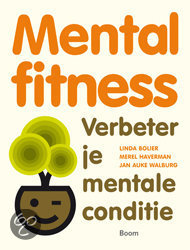


Which type of self-help would you prefer to use?

 An app on my tablet or mobile phone

 A website I can use with my PC or tablet

 A book

**3. Programme**

The self-help programme can be offered in the form of a complete course or individual modules that you can choose from. A complete course involves a number of exercises that you can follow using a computer, app or book and takes about 10 weeks to complete. Alternatively, the self-help course could involve individual modules or exercises using a website, app or book whereby you decide which modules or exercises you want to do and when.

Would you prefer to follow a complete 10-week course or choose between individual modules or exercises?

 A complete 10-week course

 Individual modules or exercises

**4. Treatment**

Various self-help methods are available for dealing with symptoms of anxiety and depression and all work differently. They can be based on cognitive behavioural therapy, problem-solving therapy, mental fitness or mindfulness. The self-help methods are explained in more detail below. The methods can be followed on a website or using a self-help book or mobile phone app.

**Cognitive behavioural therapy**

Cognitive behavioural therapy can help you to decrease the risk of a relapse of your depression or anxiety disorder. You will gain **more insight into the negative thought patterns that play a role and learn to develop a positive thought pattern, monitor your moods and prepare a prevention plan** to prevent a relapse. The following topics are discussed during the self-help course:

- changing negative thought patterns
- monitoring your mood
- thinking positive thoughts
- preparing a personal prevention plan

**Problem-solving therapy**

You will follow a self-help course which provides you with **insight into your situation and teaches you how to structure and solve problems**. This will help you to reduce feelings of stress, anxiety and/or depression. The course is intended for people who suffer from stress, anxiety, worrying, symptoms of burnout and/or depression and who want to actively do something about their problem.

The topics discussed include:

- what is really important for me in my life?
- clarifying problems by ordering them
- learning to stop worrying about unimportant matters
- learning to deal with solvable problems in small steps
- learning to accept problems that cannot be solved
- defining goals for the future

**Mental fitness**

You will follow a self-help course that trains your **mental condition**. The course includes exercises, films, tests and tips. The course will help you to **take charge of your life, live more in the here and now and deal with the pressure you feel from your environment**. You will learn what mental fitness is and how it can make you happier and more relaxed.

The course consists of the following modules:

- taking charge of your life
- mission and goals
- positive feelings
- positive relationships
- here and now
- thinking and feeling

**Mindfulness**

Mindfulness enables you to experience what is happening to you in the here and now by giving it your **full attention and without passing judgement**. The step-by-step self-help course will use **examples and mindfulness exercises to teach you to live more in the now, be more accepting of your psychological condition and live according to your own personal values**.

The mindfulness course will teach you:

- what is important to you
- how to live in the now
- what psychological distress can result in
- how to accept negative emotions and pain

Which of the above-mentioned **self-help methods** would you prefer?

 Self-help through cognitive behavioural therapy

 Self-help through problem-solving therapy

 Self-help through mental fitness

 Self-help through mindfulness

**5. Personal prevention plan**

A maintenance treatment programme could also include a personal prevention plan. This involves you and your therapist preparing a plan to prevent a relapse occurring based on your symptoms and your experiences during therapy. This plan describes the symptoms you need to be aware of, what you can do to prevent them and how you can apply the skills you learned during therapy.

Do you think a personal prevention plan should be part of the maintenance treatment programme?

 Yes

 Neutral (no opinion)

 No

**6. Time investment**

Practising skills to prevent a relapse takes time. In your previous treatment you probably also have received homework assignments. How much time are you prepared to invest in a maintenance treatment programme to prevent a relapse?

 30 minutes per week

 1 hour per week

 2 hours per week

**7. Effectiveness**

If you do nothing about your symptoms after you have finished therapy, you will have a 60% risk of suffering a relapse within four years. This risk can probably be reduced by following a maintenance treatment programme after you have finished therapy at GGZ (but will depend on the type of therapy you received).

Under what conditions would you be prepared to follow a maintenance treatment programme? (You can give more than one answer)

 If the risk of relapse decreases from 60 to 54%

 If the risk of relapse decreases from 60 to 45%

 If the risk of relapse decreases from 60 to 36%

**Specific questions about the maintenance treatment programme**

In the previous section of the questionnaire you were given information about the various parts of the maintenance treatment programme. Our aim is to develop a maintenance treatment programme that meets the needs of current and former patients as much as possible.

This section of the questionnaire discusses various maintenance treatment options. For each question, you can **choose** which treatment you would most prefer to receive. **Please answer as if you were actually going to receive this treatment after you have finished therapy at GGZ.**

You can also specify that you would prefer neither of the options. Please also specify ‘neither option’ if you would prefer to receive no treatment at all after you have finished therapy at GGZ. You can read more about the various options in the previous pages. If you have any questions you can ask the research assistant.

**Example (complete this question together with the research assistant):**

|  | **Treatment A** | **Treatment B** |
| --- | --- | --- |
| **Individual session with a therapist** | Once every 3 months | Only if you suffer a relapse |
| **Website, self-help book or app with information, exercises and tips to prevent a relapse** | Support using an app on a tablet/mobile phone | Support through a website |
| **Website/book/app programme** | Complete 10-week course | Individual modules/exercises you can choose from; unlimited |
| **Exercises (self-help)** | Self-help through cognitive behavioural therapy | Self-help through mindfulness |
| **Personal prevention plan** | The personal prevention plan is the starting point of the treatment | No personal prevention plan |
| **Time investment** | 1 hour per week | 30 minutes per week |
| **Prevention of relapse** | The risk of relapse decreases from 60 to 36% | The risk of relapse decreases from 60 to 54% |

If you were actually going to receive this treatment after you have finished therapy at GGZ, which treatment would you choose (tick appropriate box)?


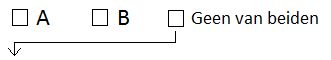


Neither option

*(If you selected neither of the options🡪)* If you had to choose, which option would you select?


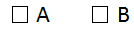


| 1. | **Treatment A** | **Treatment B** |
| --- | --- | --- |
| **Individual session with a therapist** | Once every 6 months | Once every 3 months |
| **Website, self-help book or app with information, exercises and tips to prevent a relapse** | Support using an app on a tablet/mobile phone | Support through a book |
| **Website/book/app programme** | Individual modules/exercises you can choose from; unlimited | Complete 10-week course |
| **Exercises (self-help)** | Self-help through cognitive behavioural therapy | Self-help through mental fitness |
| **Personal prevention plan** | No personal prevention plan | The personal prevention plan is the starting point of the treatment |
| **Time investment** | 2 hours per week | 1 hour per week |
| **Prevention of relapse** | The risk of relapse decreases from 60 to 45% | The risk of relapse decreases from 60 to 36% |

If you were actually going to receive this treatment after you have finished therapy at GGZ, which treatment would you choose (tick appropriate box)?


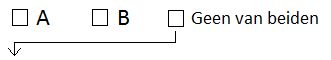


Neither option

*(If you selected neither of the options🡪)* If you had to choose, which option would you select?


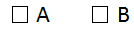


| 2. | **Treatment A** | **Treatment B** |
| --- | --- | --- |
| **Individual session with a therapist** | Only if you suffer a relapse | Once every 6 months |
| **Website, self-help book or app with information, exercises and tips to prevent a relapse** | Support through a book | Support using an app on a tablet/mobile phone |
| **Website/book/app programme** | Complete 10-week course | Individual modules/exercises you can choose from; unlimited |
| **Exercises (self-help)** | Self-help through mental fitness | Self-help through problem solving therapy |
| **Personal prevention plan** | The personal prevention plan is the starting point of the treatment | No personal prevention plan |
| **Time investment** | 2 hours per week | 1 hour per week |
| **Prevention of relapse** | The risk of relapse decreases from 60 to 45% | The risk of relapse decreases from 60 to 36% |

If you were actually going to receive this treatment after you have finished therapy at GGZ, which treatment would you choose (tick appropriate box)?


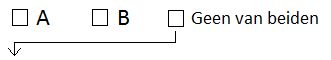


Neither option

*(If you selected neither of the options🡪)* If you had to choose, which option would you select?


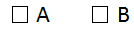


| 3. | **Treatment A** | **Treatment B** |
| --- | --- | --- |
| **Individual session with a therapist** | Once every 3 months | Only if you suffer a relapse |
| **Website, self-help book or app with information, exercises and tips to prevent a relapse** | Support through a website | Support through a book |
| **Website/book/app programme** | Complete 10-week course | Individual modules/exercises you can choose from; unlimited |
| **Exercises (self-help)** | Self-help through cognitive behavioural therapy | Self-help through problem solving therapy |
| **Personal prevention plan** | The personal prevention plan is the starting point of the treatment | No personal prevention plan |
| **Time investment** | 1 hour per week | 30 minutes per week |
| **Prevention of relapse** | The risk of relapse decreases from 60 to 54% | The risk of relapse decreases from 60 to 45% |

If you were actually going to receive this treatment after you have finished therapy at GGZ, which treatment would you choose (tick appropriate box)?


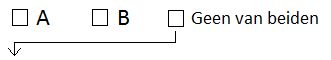


Neither option

*(If you selected neither of the options🡪)* If you had to choose, which option would you select?


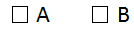


| 4. | **Treatment A** | **Treatment B** |
| --- | --- | --- |
| **Individual session with a therapist** | Once every 3 months | Once every 6 months |
| **Website, self-help book or app with information, exercises and tips to prevent a relapse** | Support using an app on a tablet/mobile phone | Support through a book |
| **Website/book/app programme** | Individual modules/exercises you can choose from; unlimited | Complete 10-week course |
| **Exercises (self-help)** | Self-help through mental fitness | Self-help through cognitive behavioural therapy |
| **Personal prevention plan** | No personal prevention plan | The personal prevention plan is the starting point of the treatment |
| **Time investment** | 30 minutes per week | 1 hour per week |
| **Prevention of relapse** | The risk of relapse decreases from 60 to 54% | The risk of relapse decreases from 60 to 45% |

If you were actually going to receive this treatment after you have finished therapy at GGZ, which treatment would you choose (tick appropriate box)?


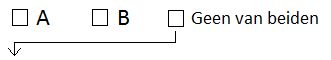


Neither option

*(If you selected neither of the options🡪)* If you had to choose, which option would you select?


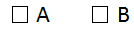


| 5. | **Treatment A** | **Treatment B** |
| --- | --- | --- |
| **Individual session with a therapist** | Only if you suffer a relapse | Once every 3 months |
| **Website, self-help book or app with information, exercises and tips to prevent a relapse** | Support using an app on a tablet/mobile phone | Support through a book |
| **Website/book/app programme** | Individual modules/exercises you can choose from; unlimited | Complete 10-week course |
| **Exercises (self-help)** | Self-help through mental fitness | Self-help through cognitive behavioural therapy |
| **Personal prevention plan** | No personal prevention plan | The personal prevention plan is the starting point of the treatment |
| **Time investment** | 1 hour per week | 2 hours per week |
| **Prevention of relapse** | The risk of relapse decreases from 60 to 45% | The risk of relapse decreases from 60 to 36% |

If you were actually going to receive this treatment after you have finished therapy at GGZ, which treatment would you choose (tick appropriate box)?


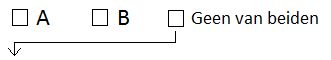


Neither option

*(If you selected neither of the options🡪)* If you had to choose, which option would you select?


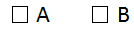


| 6. | **Treatment A** | **Treatment B** |
| --- | --- | --- |
| **Individual session with a therapist** | Only if you suffer a relapse | Once every 6 months |
| **Website, self-help book or app with information, exercises and tips to prevent a relapse** | Support through a website | Support using an app on a tablet/mobile phone |
| **Website/book/app programme** | Individual modules/exercises you can choose from; unlimited | Complete 10-week course |
| **Exercises (self-help)** | Self-help through mindfulness | Self-help through mental fitness |
| **Personal prevention plan** | The personal prevention plan is the starting point of the treatment | No personal prevention plan |
| **Time investment** | 1 hour per week | 30 minutes per week |
| **Prevention of relapse** | The risk of relapse decreases from 60 to 54% | The risk of relapse decreases from 60 to 45% |

If you were actually going to receive this treatment after you have finished therapy at GGZ, which treatment would you choose (tick appropriate box)?


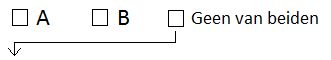


Neither option

*(If you selected neither of the options🡪)* If you had to choose, which option would you select?


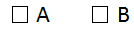


| 7. | **Treatment A** | **Treatment B** |
| --- | --- | --- |
| **Individual session with a therapist** | Once per month | Only if you suffer a relapse |
| **Website, self-help book or app with information, exercises and tips to prevent a relapse** | Support through a website | Support using an app on a tablet/mobile phone |
| **Website/book/app programme** | Complete 10-week course | Individual modules/exercises you can choose from; unlimited |
| **Exercises (self-help)** | Self-help through mindfulness | Self-help through cognitive behavioural therapy |
| **Personal prevention plan** | No personal prevention plan | The personal prevention plan is the starting point of the treatment |
| **Time investment** | 1 hour per week | 2 hours per week |
| **Prevention of relapse** | The risk of relapse decreases from 60 to 54% | The risk of relapse decreases from 60 to 45% |

If you were actually going to receive this treatment after you have finished therapy at GGZ, which treatment would you choose (tick appropriate box)?


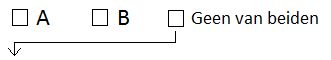


Neither option

*(If you selected neither of the options🡪)* If you had to choose, which option would you select?


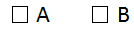


| 8. | **Treatment A** | **Treatment B** |
| --- | --- | --- |
| **Individual session with a therapist** | Once every 6 months | Once per month |
| **Website, self-help book or app with information, exercises and tips to prevent a relapse** | Support using an app on a tablet/mobile phone | Support through a website |
| **Website/book/app programme** | Complete 10-week course | Individual modules/exercises you can choose from; unlimited |
| **Exercises (self-help)** | Self-help through problem solving therapy | Self-help through mindfulness |
| **Personal prevention plan** | The personal prevention plan is the starting point of the treatment | No personal prevention plan |
| **Time investment** | 30 minutes per week | 2 hours per week |
| **Prevention of relapse** | The risk of relapse decreases from 60 to 54% | The risk of relapse decreases from 60 to 45% |

If you were actually going to receive this treatment after you have finished therapy at GGZ, which treatment would you choose (tick appropriate box)?


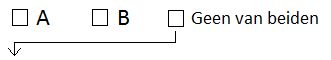


Neither option

*(If you selected neither of the options🡪)* If you had to choose, which option would you select?


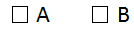


| 9. | **Treatment A** | **Treatment B** |
| --- | --- | --- |
| **Individual session with a therapist** | Only if you suffer a relapse | Once every 6 months |
| **Website, self-help book or app with information, exercises and tips to prevent a relapse** | Support using an app on a tablet/mobile phone | Support through a website |
| **Website/book/app programme** | Complete 10-week course | Individual modules/exercises you can choose from; unlimited |
| **Exercises (self-help)** | Self-help through cognitive behavioural therapy | Self-help through mental fitness |
| **Personal prevention plan** | No personal prevention plan | The personal prevention plan is the starting point of the treatment |
| **Time investment** | 1 hour per week | 1 hour per week |
| **Prevention of relapse** | The risk of relapse decreases from 60 to 54% | The risk of relapse decreases from 60 to 36% |

If you were actually going to receive this treatment after you have finished therapy at GGZ, which treatment would you choose (tick appropriate box)?


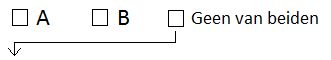


Neither option

*(If you selected neither of the options🡪)* If you had to choose, which option would you select?


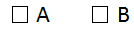


| 10. | **Treatment A** | **Treatment B** |
| --- | --- | --- |
| **Individual session with a therapist** | Once per month | Once every 3 months |
| **Website, self-help book or app with information, exercises and tips to prevent a relapse** | Support through a website | Support using an app on a tablet/mobile phone |
| **Website/book/app programme** | Individual modules/exercises you can choose from; unlimited | Complete 10-week course |
| **Exercises (self-help)** | Self-help through mental fitness | Self-help through mindfulness |
| **Personal prevention plan** | The personal prevention plan is the starting point of the treatment | No personal prevention plan |
| **Time investment** | 30 minutes per week | 2 hours per week |
| **Prevention of relapse** | The risk of relapse decreases from 60 to 45% | The risk of relapse decreases from 60 to 36% |

If you were actually going to receive this treatment after you have finished therapy at GGZ, which treatment would you choose (tick appropriate box)?


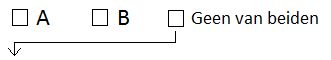


Neither option

*(If you selected neither of the options🡪)* If you had to choose, which option would you select?


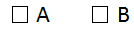


| 11. | **Treatment A** | **Treatment B** |
| --- | --- | --- |
| **Individual session with a therapist** | Once every 3 months | Only if you suffer a relapse |
| **Website, self-help book or app with information, exercises and tips to prevent a relapse** | Support through a website | Support through a book |
| **Website/book/app programme** | Individual modules/exercises you can choose from; unlimited | Complete 10-week course |
| **Exercises (self-help)** | Self-help through problem solving therapy | Self-help through mindfulness |
| **Personal prevention plan** | The personal prevention plan is the starting point of the treatment | No personal prevention plan |
| **Time investment** | 2 hours per week | 1 hour per week |
| **Prevention of relapse** | The risk of relapse decreases from 60 to 36% | The risk of relapse decreases from 60 to 54% |

If you were actually going to receive this treatment after you have finished therapy at GGZ, which treatment would you choose (tick appropriate box)?


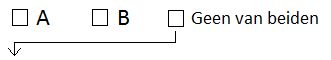


Neither option

*(If you selected neither of the options🡪)* If you had to choose, which option would you select?


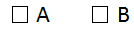


| 12. | **Treatment A** | **Treatment B** |
| --- | --- | --- |
| **Individual session with a therapist** | Once every 6 months | Once every 3 months |
| **Website, self-help book or app with information, exercises and tips to prevent a relapse** | Support through a book | Support through a website |
| **Website/book/app programme** | Individual modules/exercises you can choose from; unlimited | Complete 10-week course |
| **Exercises (self-help)** | Self-help through mental fitness | Self-help through problem solving therapy |
| **Personal prevention plan** | The personal prevention plan is the starting point of the treatment | No personal prevention plan |
| **Time investment** | 2 hours per week | 1 hour per week |
| **Prevention of relapse** | The risk of relapse decreases from 60 to 36% | The risk of relapse decreases from 60 to 45% |

If you were actually going to receive this treatment after you have finished therapy at GGZ, which treatment would you choose (tick appropriate box)?


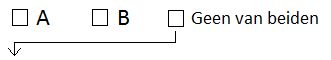


Neither option

*(If you selected neither of the options🡪)* If you had to choose, which option would you select?


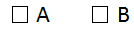


| 13. | **Treatment A** | **Treatment B** |
| --- | --- | --- |
| **Individual session with a therapist** | Only if you suffer a relapse | Once every 6 months |
| **Website, self-help book or app with information, exercises and tips to prevent a relapse** | Support using an app on a tablet/mobile phone | Support through a website |
| **Website/book/app programme** | Complete 10-week course | Individual modules/exercises you can choose from; unlimited |
| **Exercises (self-help)** | Self-help through problem solving therapy | Self-help through cognitive behavioural therapy |
| **Personal prevention plan** | The personal prevention plan is the starting point of the treatment | No personal prevention plan |
| **Time investment** | 1 hour per week | 30 minutes per week |
| **Prevention of relapse** | The risk of relapse decreases from 60 to 36% | The risk of relapse decreases from 60 to 54% |

If you were actually going to receive this treatment after you have finished therapy at GGZ, which treatment would you choose (tick appropriate box)?


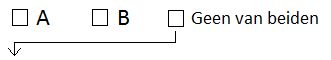


Neither option

*(If you selected neither of the options🡪)* If you had to choose, which option would you select?


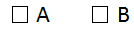


| 14. | **Treatment A** | **Treatment B** |
| --- | --- | --- |
| **Individual session with a therapist** | Once every 6 months | Once per month |
| **Website, self-help book or app with information, exercises and tips to prevent a relapse** | Support through a book | Support using an app on a tablet/mobile phone |
| **Website/book/app programme** | Complete 10-week course | Individual modules/exercises you can choose from; unlimited |
| **Exercises (self-help)** | Self-help through mindfulness | Self-help through mental fitness |
| **Personal prevention plan** | No personal prevention plan | No personal prevention plan |
| **Time investment** | 1 hour per week | 2 hours per week |
| **Prevention of relapse** | The risk of relapse decreases from 60 to 36% | The risk of relapse decreases from 60 to 45% |

If you were actually going to receive this treatment after you have finished therapy at GGZ, which treatment would you choose (tick appropriate box)?


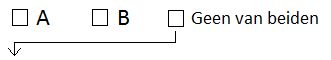


Neither option

*(If you selected neither of the options🡪)* If you had to choose, which option would you select?


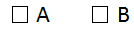


| 15. | **Treatment A** | **Treatment B** |
| --- | --- | --- |
| **Individual session with a therapist** | Once per month | Once every 3 months |
| **Website, self-help book or app with information, exercises and tips to prevent a relapse** | Support through a book | Support using an app on a tablet/mobile phone |
| **Website/book/app programme** | Individual modules/exercises you can choose from; unlimited | Complete 10-week course |
| **Exercises (self-help)** | Self-help through problem solving therapy | Self-help through mindfulness |
| **Personal prevention plan** | No personal prevention plan | The personal prevention plan is the starting point of the treatment |
| **Time investment** | 2 hours per week | 30 minutes per week |
| **Prevention of relapse** | The risk of relapse decreases from 60 to 36% | The risk of relapse decreases from 60 to 45% |

If you were actually going to receive this treatment after you have finished therapy at GGZ, which treatment would you choose (tick appropriate box)?


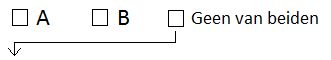


Neither option

*(If you selected neither of the options🡪)* If you had to choose, which option would you select?


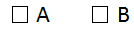


| 16. | **Treatment A** | **Treatment B** |
| --- | --- | --- |
| **Individual session with a therapist** | Once per month | Only if you suffer a relapse |
| **Website, self-help book or app with information, exercises and tips to prevent a relapse** | Support through a book | Support through a website |
| **Website/book/app programme** | Complete 10-week course | Individual modules/exercises you can choose from; unlimited |
| **Exercises (self-help)** | Self-help through problem solving therapy | Self-help through mental fitness |
| **Personal prevention plan** | The personal prevention plan is the starting point of the treatment | The personal prevention plan is the starting point of the treatment |
| **Time investment** | 30 minutes per week | 1 hour per week |
| **Prevention of relapse** | The risk of relapse decreases from 60 to 45% | The risk of relapse decreases from 60 to 54% |

If you were actually going to receive this treatment after you have finished therapy at GGZ, which treatment would you choose (tick appropriate box)?


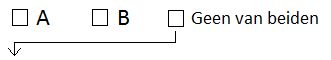


Neither option

*(If you selected neither of the options🡪)* If you had to choose, which option would you select?


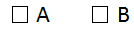


| 17. | **Treatment A** | **Treatment B** |
| --- | --- | --- |
| **Individual session with a therapist** | Once every 3 months | Once per month |
| **Website, self-help book or app with information, exercises and tips to prevent a relapse** | Support through a book | Support using an app on a tablet/mobile phone |
| **Website/book/app programme** | Complete 10-week course | Individual modules/exercises you can choose from; unlimited |
| **Exercises (self-help)** | Self-help through cognitive behavioural therapy | Self-help through mindfulness |
| **Personal prevention plan** | No personal prevention plan | The personal prevention plan is the starting point of the treatment |
| **Time investment** | 1 hour per week | 2 hours per week |
| **Prevention of relapse** | The risk of relapse decreases from 60 to 45% | The risk of relapse decreases from 60 to 36% |

If you were actually going to receive this treatment after you have finished therapy at GGZ, which treatment would you choose (tick appropriate box)?


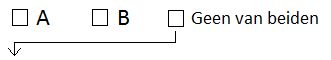


Neither option

*(If you selected neither of the options🡪)* If you had to choose, which option would you select?


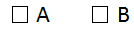


| 18. | **Treatment A** | **Treatment B** |
| --- | --- | --- |
| **Individual session with a therapist** | Once every 6 months | Once per month |
| **Website, self-help book or app with information, exercises and tips to prevent a relapse** | Support through a website | Support through a book |
| **Website/book/app programme** | Complete 10-week course | Individual modules/exercises you can choose from; unlimited |
| **Exercises (self-help)** | Self-help through mindfulness | Self-help through cognitive behavioural therapy |
| **Personal prevention plan** | The personal prevention plan is the starting point of the treatment | No personal prevention plan |
| **Time investment** | 2 hours per week | 30 minutes per week |
| **Prevention of relapse** | The risk of relapse decreases from 60 to 45% | The risk of relapse decreases from 60 to 54% |

If you were actually going to receive this treatment after you have finished therapy at GGZ, which treatment would you choose (tick appropriate box)?


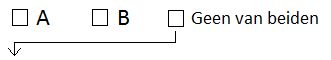


Neither option

*(If you selected neither of the options🡪)* If you had to choose, which option would you select?


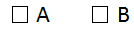


| 19. | **Treatment A** | **Treatment B** |
| --- | --- | --- |
| **Individual session with a therapist** | Once per month | Only if you suffer a relapse |
| **Website, self-help book or app with information, exercises and tips to prevent a relapse** | Support using an app on a tablet/mobile phone | Support through a book |
| **Website/book/app programme** | Individual modules/exercises you can choose from; unlimited | Complete 10-week course |
| **Exercises (self-help)** | Self-help through cognitive behavioural therapy | Self-help through problem solving therapy |
| **Personal prevention plan** | The personal prevention plan is the starting point of the treatment | No personal prevention plan |
| **Time investment** | 1 hour per week | 30 minutes per week |
| **Prevention of relapse** | The risk of relapse decreases from 60 to 36% | The risk of relapse decreases from 60 to 54% |

If you were actually going to receive this treatment after you have finished therapy at GGZ, which treatment would you choose (tick appropriate box)?


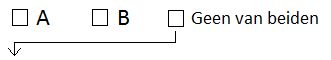


Neither option

*(If you selected neither of the options🡪)* If you had to choose, which option would you select?


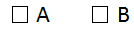


| 20. | **Treatment A** | **Treatment B** |
| --- | --- | --- |
| **Individual session with a therapist** | Once every 3 months | Once per month |
| **Website, self-help book or app with information, exercises and tips to prevent a relapse** | Support through a book | Support through a website |
| **Website/book/app programme** | Individual modules/exercises you can choose from; unlimited | Complete 10-week course |
| **Exercises (self-help)** | Self-help through mindfulness | Self-help through problem solving therapy |
| **Personal prevention plan** | No personal prevention plan | The personal prevention plan is the starting point of the treatment |
| **Time investment** | 30 minutes per week | 1 hour per week |
| **Prevention of relapse** | The risk of relapse decreases from 60 to 45% | The risk of relapse decreases from 60 to 54% |

If you were actually going to receive this treatment after you have finished therapy at GGZ, which treatment would you choose (tick appropriate box)?


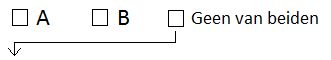


Neither option

*(If you selected neither of the options🡪)* If you had to choose, which option would you select?


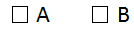


If you have any comments about this questionnaire, please enter these below.

**Please continue to the next page to complete the last section of the questionnaire. This section contains questions about your health, your symptoms and your daily functioning.**

[A. WHO Disability Assessment Schedule (WHODAS) [50]]

[B. Inventory of Depressive Symptomatology (IDS) [51]]

[C. Beck Anxiety Inventory (BAI) [52]]

[D. Anxiety Sensitivity Index (ASI) [53]]
