# Supplementary material for: Patient preferences for a guided self-help programme to prevent relapse in anxiety or depression: A discrete choice experiment
Source: PLoS One. 2019 Jul 18;14(7):e0219588. doi: 10.1371/journal.pone.0219588 (PMC6638925; doi:10.1371/journal.pone.0219588)
Supplement: S4 File — (DOCX) [file pone.0219588.s007.docx]

**Supplement 4. Experimental design (NGene syntax)**

??????????????????????????????????????

?

? Initial D-efficient design

?

????? cf = contact frequency

? 0=only when needed

? 1=every 6 months

? 2= every 3 months

? 3=every month

????? dm = delivery mode

? 0=book

? 1=website

? 2=app

????? pf = program flexibility

? 0= complete 10 week course

? 1= individual modules or exercises

????? tt=treatment type

? 0=cognitive behavioural therapy

? 1= problem solving therapy

? 2=positive psychology

? 3=Mindfulness

????? pp = personal prevention plan

? 0=not included in intervention

? 1= included in intervention

????? ti = time investment

? 0=0.5 hour per week

? 1=1 hour per week

? 2=2 hours per week

????? ef = effectiveness

? 0= decrease in risk of relapse from 60% to 54%

? 1= decrease in risk of relapse from 60% to 45%

? 2= decrease in risk of relapse from 60% to 36%

?

Design

? Three alternatives

;alts=alt1, alt2, alt0

? Twenty choice sets

;rows=20

? Create a D-efficient design

;eff=(mnl,d)

? Mandatory combinations to exclude undesirable combinations

;cond:

if (alt1.time_investment=0, alt1.effectiveness=[1,2]),

if (alt1.time_investment=2, alt1.effectiveness=[0,1]),

if (alt2.time_investment=0, alt2.effectiveness=[1,2]),

if (alt2.time_investment=2, alt2.effectiveness=[0,1])

? Utility functions

;model:

U(alt1) = b0_const

+ b1_cf.dummy[0.0|0.0|0.0] * contact_frequency[1,2,3,0]

+ b2_dm.dummy[0.0|0.0] * delivery_mode[1,2,0]

+ b3_pf[0.0] * program_flexibility[1,0]

+ b4_tt.dummy[0.0|0.0|0.0] * treatment_type[1,2,3,0]

+ b5_pp[0.0] * personal_prevention_plan[1,0]

+ b6_ti.dummy[-0.05|-0.10] * time_investment[1,2,0]

+ b7_ef.dummy[0.1|0.2] * effectiveness[1,2,0] /

U(alt2) = b1_cf * contact_frequency

+ b2_dm * delivery_mode

+ b3_of * program_flexibility

+ b4_tt * treatment_type

+ b5_pp * personal_prevention_plan

+ b6_ti * time_investment

+ b7_ef * effectiveness

$

??????????????????????????????????????

? Final Bayesian D-efficient design

?

????? cf = contact frequency

? 0=only when needed

? 1=every 6 months

? 2= every 3 months

? 3=every month

????? dm = delivery mode

? 0=book

? 1=web site

? 2=app

????? pf = program flexibility

? 0= complete 10-week course

? 1= individual modules and exercises

????? tt=treatment type

? 0=cognitive behavioural therapy

? 1= problem solving therapy

? 2=positive psychology

? 3=Mindfulness

????? pp = personal prevention plan

? 0=not included in intervention

? 1= included in intervention

????? ti = time investment

? 0=0.5 hour per week

? 1=1 hour per week

? 2=2 hours per week

????? ef = effectiveness

? 0= decrease in risk of relapse from 60% to 54%

? 1= decrease in risk of relapse from 60% to 45%

? 2= decrease in risk of relapse from 60% to 36%

?

Design

? Three alternatives

;alts=alt1, alt2, alt0

? Twenty choice sets

;rows=20

? Create a D-efficient design

;eff=(mnl,d)

? Mandatory combinations to exclude undesirable combinations

;cond:

if (alt1.time_investment=0, alt1.effectiveness=[1,2]),

if (alt1.time_investment=2, alt1.effectiveness=[0,1]),

if (alt2.time_investment=0, alt2.effectiveness=[1,2]),

if (alt2.time_investment=2, alt2.effectiveness=[0,1])

? Utility functions

;model:

U(alt1) = b0_const

+ b1_cf.dummy[(n,0.1,0.2)|(n,0.3,0.2)|(n,0.15,0.2)]

* contact_frequency[1,2,3,0]

+ b2_dm.dummy[(n,0.0,0.2)|(n,0.0,0.2)] * delivery_mode[1,2,0]

+ b3_vl[(n,0.2,0.1)] * program_flexibility[1,0]

+ b4_tt.dummy[(n,0.0,0.2)|(n,-0.2,0.2)|(n,-0.1,0.2)]

* treatment_type[1,2,3,0]

+ b5_pp[(n,0.6,0.15)] * personal_prevention_plan[1,0]

+ b6_ti.dummy[(n,-0.3,0.15)|(n,-0.4,0.2)] * time_investment[1,2,0]

+ b7_ef.dummy[(n,0.0,0.2)|(n,0.4,0.2)] * effectiveness[1,2,0] /

U(alt2) = b1_cf * contact_frequency

+ b2_dm * delivery_mode

+ b3_of * program_flexibility

+ b4_tt * treatment_type

+ b5_pp * personal_prevention_plan

+ b6_ti * time_investment

+ b7_ef * effectiveness

$
