# Supplementary material for: Patient preferences for a guided self-help programme to prevent relapse in anxiety or depression: A discrete choice experiment
Source: PLoS One. 2019 Jul 18;14(7):e0219588. doi: 10.1371/journal.pone.0219588 (PMC6638925; doi:10.1371/journal.pone.0219588)
Supplement: S5 File — (DOCX) [file pone.0219588.s008.docx]

S5 File – Final survey including DCE [in Dutch]

**Vragenlijst voorkeuren voor ondersteuning na afsluiten van behandeling**

Uit onderzoek blijkt dat mensen die een angststoornis of een depressie gehad hebben een verhoogd risico lopen om opnieuw klachten te krijgen. Ongeveer 1 op de 4 patiënten die hersteld zijn, krijgt binnen 2 jaar opnieuw klachten. Binnen 4 jaar krijgt zelfs 60% van de patiënten opnieuw een angst- of depressieve stoornis. Het volgen van een onderhoudsbehandeling verkleint het risico om opnieuw klachten te krijgen. Er is echter nog geen onderzoek gedaan naar hoe patiënten ondersteund willen worden na afsluiten van hun behandeling in de GGZ.

Met dit onderzoek willen we de wensen en meningen van (voormalig) patiënten ten opzichte van een onderhoudsbehandeling in kaart brengen. Deze vragenlijst is bedoeld om meer inzicht te krijgen welke ondersteuning u nodig denkt te hebben na de afronding van uw therapie. In deze vragenlijst wordt daarom naar uw mening gevraagd over verschillende onderhoudsbehandelingen. De gegevens die u invult worden strikt vertrouwelijk behandeld. Uw deelname aan het onderzoek heeft geen gevolgen voor uw behandeling.

U begint met een aantal algemene vragen. Daarna leest u eerst informatie over verschillende mogelijke onderhoudsbehandelingen. U geeft aan welke onderhoudsbehandeling u het meest aanspreekt. Ten slotte vult u vier vragenlijsten in die gaan over de angst- en/of depressieve klachten die u op *dit* moment ervaart.

Het duurt ongeveer 60 minuten om alle vragenlijsten in te vullen. Voor vragen kunt u terecht bij de onderzoeksassistent. Alvast hartelijk bedankt dat u wilt meewerken aan dit onderzoek!

Mw. dr. A. Muntingh, onderzoeker GGZ inGeest

**Persoonsgegevens**

1. Datum: (dag/maand/jaar) ……./…../…………
2. Geslacht:  Man  Vrouw
3. Wat is uw leeftijd? …….. jaar
4. Voor welke klachten bent/was u onlangs in behandeling? (meerdere antwoorden mogelijk)

 Angstklachten

 Depressieve klachten

1. Welke type behandeling heeft u gevolgd voor uw klachten? (meerdere antwoorden mogelijk)

 cognitieve gedragstherapie

 interpersoonlijke therapie

 medicatie

 anders, namelijk……………………………………………………………………

S

1. Bent u eerder in behandeling geweest voor uw klachten bij een psycholoog, psychiater of GGZ-instelling?

 ja, namelijk ……. (aantal) keer

 nee

1. Heeft u ooit zelfhulp en/of behandeling via internet gevolgd voor uw depressieve- of angstklachten? (meerdere antwoorden mogelijk)

 Zelfhulp

 Internetbehandeling

 Geen van beiden

1. Op welke leeftijd had u voor het eerst een depressie of angststoornis?

|  |
| --- |

Leeftijd:

1. Wat is uw hoogst afgeronde opleiding? (omcirkel één antwoord)

1. Lagere school, basisonderwijs

2. Lager onderwijs of voorbereidend beroepsonderwijs(LBO, LTS, Huishoudschool)

3. Middelbaar algemeen onderwijs Mavo, Mulo, Ulo, VMBO

4. Middelbaar beroepsonderwijs (MBO, MTS, MEAO)

5. Voortgezet algemeen onderwijs (Havo/VWO/Gymnasium/HBS/MMS)

6. Hoger beroepsonderwijs (HTS, HEAO, HHNO)of Wetenschappelijk onderwijs (WO)

7. Anders namelijk …..

1. Heeft u een naast familielid (broer/zus/ouder/kind) met een depressie of een angststoornis?

 nee, geen psychologische klachten binnen mijn familie *(ga door naar vraag 11)*

 ja, er zijn psychologische klachten binnen mijn familie.

*Vul in welke type stoornis (depressie of angststoornis) en bij welk familielid:*

1. (type stoornis)……………………………………………………………bij (familielid)……….......
2. (type stoornis)……………………………………………………………bij (familielid)……….......
3. (type stoornis)…………………………………………………………….bij (familielid)……….......
4. (type stoornis)…………………………………………………………….bij (familielid)……….......
5. **Hoeveel risico** denkt u dat u loopt om opnieuw een angst- of depressieve stoornis te krijgen in de komende 4 jaar? (zet een kruisje op onderstaande lijn)

|----------------------------------------------------------------------------------------------|
0% 10% 20% 30% 40% 50% 60% 70% 80% 90% 100%

**Uitleg bij de onderdelen van de verschillende onderhoudsbehandelingen**

Uit onderzoek blijkt dat mensen die een angststoornis of een depressie gehad hebben en die een onderhoudsbehandeling krijgen ná afronding van de therapie, minder risico lopen om opnieuw klachten te krijgen. De onderhoudsbehandelingen die in dit onderzoek worden onderzocht bestaan uit **persoonlijk contact** met een psychiatrisch verpleegkundige of psycholoog in de huisartspraktijk, aangevuld met informatie en oefeningen via een zelfhulpboek of een website. Wij willen onderzoeken hoe zo’n onderhoudsbehandeling er precies uit moet zien volgens (voormalig)patiënten. Uw mening speelt dus een belangrijke rol bij het ontwikkelen van een nieuwe onderhoudsbehandeling. Neemt u rustig de tijd om de informatie hieronder door te lezen. De onderzoeksassistent helpt u bij het invullen van de vragen.

Als u een onderhoudsbehandeling krijgt aangeboden na afsluiten van uw behandeling, waarbij u persoonlijk contact heeft met een psycholoog of psychiatrisch verpleegkundige in de huisartspraktijk en thuis aan de slag gaat met zelfhulp, gaat u dit dan doen?

 Ja, ik denk het wel

 Nee, ik denk het niet

**Onderdelen van de (toekomstige) onderhoudsbehandeling**

**1. De frequentie van de gesprekken met een behandelaar in de huisartsenpraktijk.**

Een onderdeel van de onderhoudsbehandeling bestaat uit een persoonlijk contact (face-to-face) in de vorm van gesprekken met een behandelaar (psycholoog of psychiatrisch verpleegkundige) die werkt in de huisartspraktijk. In deze gesprekken bespreekt u hoe het met u gaat. De behandelaar kan u ook helpen bij eventuele zelfhulp die u volgt.

Stel dat u moest kiezen hoe vaak u uw behandelaar zou spreken (face-to-face), hoe vaak zou dan een gesprek willen hebben met uw behandelaar?

 1x per maand

 1x per 3 maanden

 1x per 6 maanden

 Alleen wanneer ik weer klachten krijg

**2. De vorm van zelfhulp**

Zelfhulp vormt, naast het persoonlijk contact met uw behandelaar, een belangrijk onderdeel bij onderhoudsbehandelingen. Met zelfhulp kunt u zelf terugval voorkomen door te blijven werken aan uw vaardigheden om met (terugkerende) klachten om te gaan. Er zijn verschillende vormen van zelfhulp. Bij de zelfhulp wordt u ondersteund door uw behandelaar op de huisartspraktijk.

- Via een **App** op uw mobiele telefoon. Deze app geeft informatie, opdrachten en oefeningen voor het omgaan met angst- of depressieve klachten.

*Voorbeeld van een oefening uit de app:*

- Via een **internetsite**. U krijgt toegang tot uw gepersonaliseerde pagina waarin u met gebruikersnaam en wachtwoord inlogt. Op de website vindt u informatie, opdrachten en oefeningen voor het omgaan met angst- of depressieve klachten.

*Voorbeeld van een internetbehandeling*


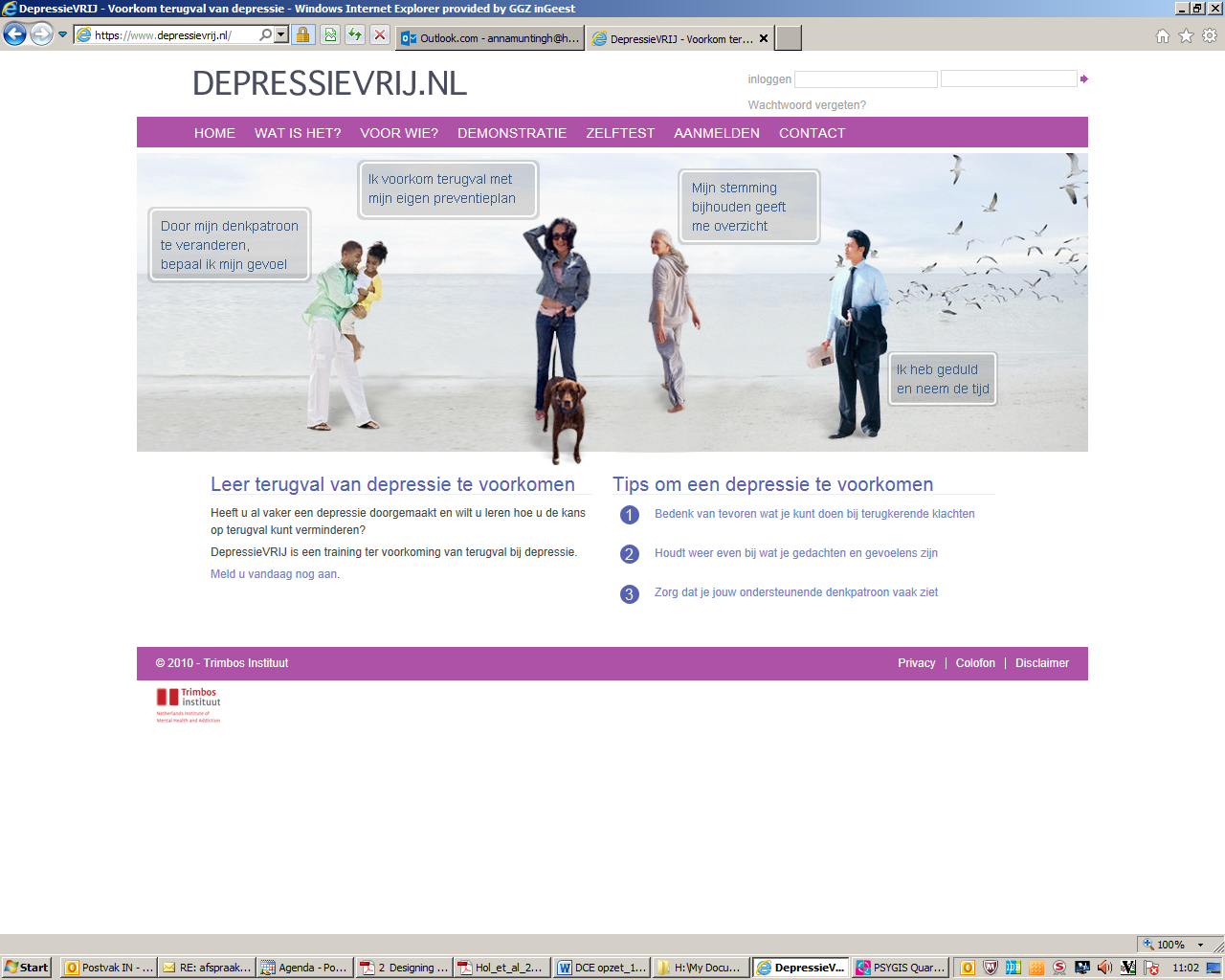


Leer terugval te voorkomen

Heeft u een depressie of angststoornis doorgemaakt en wit u leren hoe u de kans op terugval kunt verminderen? Volg dan deze training ter voorkoming van terugval bij depressie en angst.

Tips om terugval te voorkomen

- Via een boek. U schaft een zelfhulpboek aan die u stap voor stap op eigen tempo doorwerkt. In het dit boek staan tips en adviezen over hoe u uw klachten kunt aanpakken. Uw behandelaar ondersteunt u in het gebruik van het zelfhulpboek.

*Voorbeeld van een boek*


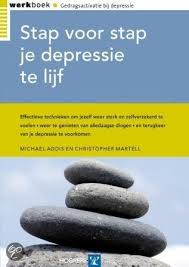


- Via een **zelfhulpboek**. U krijgt een boek met informatie, opdrachten en oefeningen voor het omgaan met angst- of depressieve klachten.


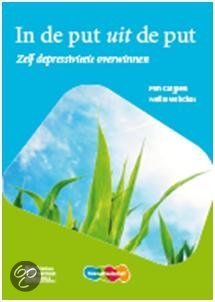
*Voorbeelden van zelfhulpboeken*

*
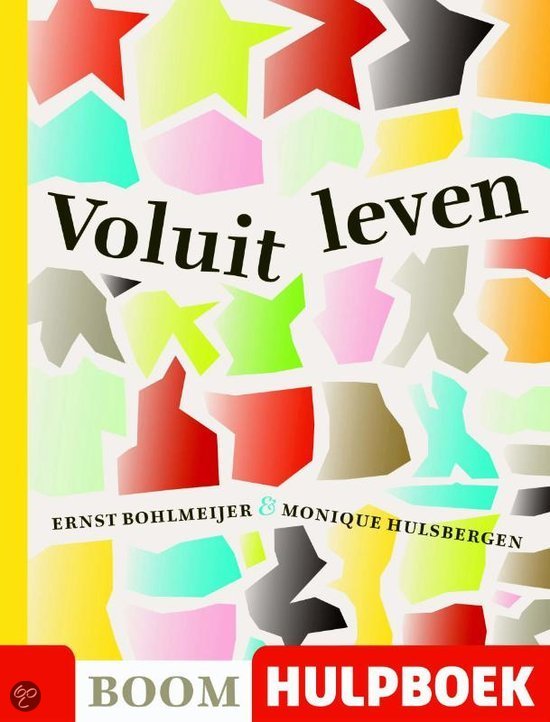
*
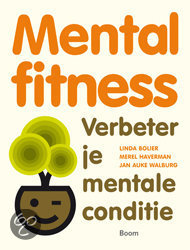


Als u gebruik zou maken van zelfhulp, welke vorm van zelfhulp zou u dan kiezen?

 Een app op mijn tablet of mobiele telefoon

 Een website voor PC of tablet

 Een boek

**3. Programma**

De zelfhulp kan bestaan uit een vast programma of uit losse onderdelen die u zelf kiest. Bij een vast programma moet u denken aan een aantal “lessen” die u volgt via de computer, app of een boek en die u in ongeveer 10 weken doorwerkt. De zelfhulp kan ook bestaan uit een website, app of boek met losse modules of oefeningen waar u steeds uit kunt kiezen, zonder vaste volgorde.

Stel dat u zou moeten kiezen tussen een vast programma van 10 weken of losse onderdelen, op welke manier u de zelfhulp aangeboden willen krijgen?

 Een vast programma van 10 weken

 Losse modules of oefeningen

**4. Inhoud behandelingen**

Er bestaan verschillende zelfhulpmethodes voor het omgaan met angst- en depressieve klachten. De inhoud van deze zelfhulpmethodes verschilt. Ze zijn gebaseerd op cognitieve gedragstherapie, probleemoplossende therapie, mentale fitheid of mindfulness. Hieronder volgt uitleg over de verschillende zelfhulpmethodes. U kunt deze methodes volgen via internet, met een zelfhulpboek of via een app op uw mobiele telefoon.

**Cognitieve gedragstherapie**

Met behulp van cognitieve gedragstherapie kunt u de kans op een terugkerende depressie of angststoornis verminderen. U krijgt een **beter inzicht in de denkpatronen die bij u een rol spelen, u leert een passend denkpatroon te ontwikkelen, de stemming te monitoren en een preventieplan op te stellen** om terugval te voorkomen. In deze zelfhulpcursus komen de volgende onderwerpen aan bod:

- Het veranderen van denkpatronen
- Bijhouden van de stemming
- Positieve gebeurtenissen onthouden
- Opstellen van een persoonlijk preventieplan

**Probleemoplossende therapie**

U volgt een zelfhulpcursus waarin u **inzicht krijgt in uw situatie en problemen leert ordenen en aanpakken**. Hiermee kunt u klachten van stress, angst en/of somberheid verminderen. De cursus is bedoeld voor mensen die last hebben van stress, piekeren, angstgevoelens, een opgebrand gevoel en/of somberheid en hier actief mee aan de slag willen.

Onderwerpen die aan de orde komen zijn:

- Wat is werkelijk belangrijk in mijn leven?
- Problemen op een rijtje zetten en ordenen
- Leren stoppen met piekeren over onbelangrijke zaken
- Leren oplosbare problemen in kleine stappen aan te pakken
- Acceptatiemethodes voor onoplosbare problemen
- Doelen voor de toekomst bedenken

**Mentale fitheid**

U volgt een zelfhulpcursus waarmee u uw **mentale conditie** traint. Dit doet u met oefeningen, filmpjes, tests en tips. Na het volgen van de cursus **heeft u meer grip op uw leven, leeft u meer in het hier en nu en kunt u beter omgaan met de druk die de omgeving op u legt**. In de cursus leert u wat mentale fitheid is en hoe u gelukkiger en meer ontspannen kunt leven.

De cursus bestaat uit de volgende modules:

- Grip op je leven
- Missie en doelen
- Positief gevoel
- Positieve relaties
- Hier en nu
- Denken en voelen

**Mindfulness**

Mindfulness is het vermogen om **aandachtig en zonder oordeel** op te merken wat zich in het hier en nu voordoet. In deze zelfhulpcursus leert u aan de hand van **voorbeelden en mindfulness oefeningen stapsgewijs om met aandacht in het nu, zonder verzet tegen psychisch leed en vanuit persoonlijke waarden te leven.**

In de cursus Mindfulness leert u:

- wat voor u waardevol is.
- met aandacht te leven in het nu.
- wat psychisch leed veroorzaakt.
- milder te worden naar uw negatieve emoties of pijn.

Als u moest kiezen tussen het volgen van de bovenstaande **zelfhulpmethodes**, welke zou u dan kiezen?

 Zelfhulp gebaseerd op cognitieve gedragstherapie

 Zelfhulp gebaseerd op probleem oplossende therapie

 Zelfhulp gebaseerd op mentale fitheid

 Zelfhulp gebaseerd op mindfulness

**5. Persoonlijk Preventieplan**

Een persoonlijk preventieplan kan onderdeel zijn van de onderhoudsbehandeling. U stelt met uw behandelaar een plan op om terugval te voorkómen, aan de hand van uw klachten en uw ervaringen in de therapie. In dit plan staan de signalen die bij u op terugval kunnen wijzen, wat u dan kunt doen en welke vaardigheden die u in de therapie geleerd heeft u opnieuw kunt inzetten.

Vindt u dat een persoonlijk preventieplan onderdeel uitgangspunt moet zijn van de onderhoudsbehandeling?

 Ja

 Neutraal (maakt niet uit)

 Nee

**6. Tijdsinvestering**

Het oefenen met vaardigheden om terugval te voorkómen, kost tijd. Waarschijnlijk heeft u tijdens uw behandelingen ook thuis huiswerkopdrachten gemaakt.

Hoeveel tijd bent u bereid te investeren in een onderhoudsbehandeling om terugval in de klachten te voorkómen?

 ½ uur per week

 1 uur per week

 2 uur per week

**7. Effectiviteit**

Als u na uw behandeling stopt met het werken aan uw klachten, loopt u 60% kans dat u binnen 4 jaar opnieuw klachten krijgt. Uw risico op terugval als u niets doet is dus 60%. Dit risico kan waarschijnlijk worden verkleind als u een onderhoudsbehandeling volgt na afsluiten van de behandeling in de GGZ. Afhankelijk van het type behandeling wordt het risico op terugval kleiner.

Wanneer bent u bereid om een onderhoudsbehandeling te volgen? (meerdere antwoorden mogelijk)

 Als het risico op terugval afneemt van 60 naar 54%

 Als het risico op terugval afneemt van 60 naar 45%

 Als het risico op terugval afneemt van 60 naar 36%

**Specifieke vragen over de verschillende onderhoudsbehandelingen**

In het vorige deel van de vragenlijst heeft u informatie gekregen over verschillende onderdelen van onderhoudsbehandelingen. Wij proberen een onderhoudsbehandeling te ontwikkelen die het meest aansluit bij de voorkeuren van (voormalig)patiënten.

In dit deel van de vragenlijst worden verschillende onderhoudsbehandelingen vergeleken. Bij elke vraag maakt u een **keuze** voor de behandeling die u het liefst zou willen volgen. **Stel u voor dat u deze behandeling daadwerkelijk gaat volgen na het afsluiten van de behandeling bij de GGZ.**

U kunt ook aangeven dat u geen van beide behandelingen zou willen volgen. Ook als u helemaal geen onderhoudsbehandeling wilt volgen na afsluiten van uw behandeling in de GGZ, kunt u de optie geen van beiden aankruisen. De informatie over de onderdelen kunt u teruglezen op de vorige pagina’s. Voor vragen kunt u terecht bij de onderzoeksassistent.

**Voorbeeld (vul deze vraag in met de onderzoeksassistente):**

|  | **Behandeling A** | **Behandeling B** |
| --- | --- | --- |
| **Gesprek met een behandelaar** | 1x per 3 maanden | Alleen wanneer u weer klachten krijgt |
| **Website, zelfhulpboek of app met informatie, oefeningen en tips om terugval te voorkómen** | Ondersteuning via App op iPad / mobiele telefoon | Ondersteuning via website |
| **Inhoud website / boek / app** | Vast programma van 10 weken | Losse onderdelen/oefeningen waar u uit kunt kiezen, doorlopend |
| **Inhoud oefeningen (zelfhulp)** | Zelfhulp gebaseerd op cognitieve gedragstherapie | Zelfhulp gebaseerd op mindfulness |
| **Persoonlijk preventieplan** | Persoonlijk preventieplan is uitgangspunt van de behandeling | U heeft geen persoonlijk preventieplan |
| **Tijdsinvestering** | 1 uur per week | ½ uur per week |
| **Bescherming tegen terugval** | Uw risico op terugval neemt af van 60% naar 36% | Uw risico op terugval neemt af van 60% naar 54% |

Stel u voor dat u deze behandeling krijgt aangeboden na het afsluiten van de behandeling bij de GGZ. Welke behandeling kiest u (kruis aan)?


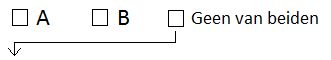


*(Wanneer u geen van beiden heeft ingevuld 🡪)* Als u moest kiezen, welke behandeling zou u dan kiezen?


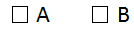


| **1.** | **Behandeling A** | **Behandeling B** |
| --- | --- | --- |
| **Gesprek met een behandelaar** | 1x per 6 maanden | 1x per 3 maanden |
| **Website, zelfhulpboek of app met informatie, oefeningen en tips om terugval te voorkómen** | Ondersteuning via App op iPad / mobiele telefoon | Ondersteuning via zelfhulpboek |
| **Inhoud website / boek / app** | Losse onderdelen/oefeningen waar u uit kunt kiezen, doorlopend | Vast programma van 10 weken |
| **Inhoud oefeningen (zelfhulp)** | Zelfhulp gebaseerd op cognitieve gedragstherapie | Zelfhulp gebaseerd op mentale fitheid |
| **Persoonlijk preventieplan** | U heeft geen persoonlijk preventieplan | Persoonlijk preventieplan is uitgangspunt van de behandeling |
| **Tijdsinvestering** | 2 uur per week | 1 uur per week |
| **Bescherming tegen terugval** | Uw risico op terugval neemt af van 60% naar 45% | Uw risico op terugval neemt af van 60% naar 36% |

Stel u voor dat u deze behandeling krijgt aangeboden na het afsluiten van de behandeling bij de GGZ. Welke behandeling kiest u (kruis aan)?


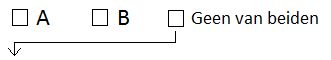


*(Wanneer u geen van beiden heeft ingevuld 🡪)* Als u moest kiezen, welke behandeling zou u dan kiezen?


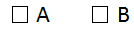


| **2.** | **Behandeling A** | **Behandeling B** |
| --- | --- | --- |
| **Gesprek met een behandelaar** | Alleen wanneer u weer klachten krijgt | 1x per 6 maanden |
| **Website, zelfhulpboek of app met informatie, oefeningen en tips om terugval te voorkómen** | Ondersteuning via zelfhulpboek | Ondersteuning via App op iPad / mobiele telefoon |
| **Inhoud website / boek / app** | Vast programma van 10 weken | Losse onderdelen/oefeningen waar u uit kunt kiezen, doorlopend |
| **Inhoud oefeningen (zelfhulp)** | Zelfhulp gebaseerd op mentale fitheid | Zelfhulp gebaseerd op probleem oplossende therapie |
| **Persoonlijk preventieplan** | Persoonlijk preventieplan is uitgangspunt van de behandeling | U heeft geen persoonlijk preventieplan |
| **Tijdsinvestering** | 2 uur per week | 1 uur per week |
| **Bescherming tegen terugval** | Uw risico op terugval neemt af van 60% naar 45% | Uw risico op terugval neemt af van 60% naar 36% |

Stel u voor dat u deze behandeling krijgt aangeboden na het afsluiten van de behandeling bij de GGZ. Welke behandeling kiest u (kruis aan)?


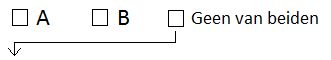


*(Wanneer u geen van beiden heeft ingevuld 🡪)* Als u moest kiezen, welke behandeling zou u dan kiezen?


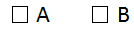


| **3.** | **Behandeling A** | **Behandeling B** |
| --- | --- | --- |
| **Gesprek met een behandelaar** | 1x per 3 maanden | Alleen wanneer u weer klachten krijgt |
| **Website, zelfhulpboek of app met informatie, oefeningen en tips om terugval te voorkómen** | Ondersteuning via website | Ondersteuning via zelfhulpboek |
| **Inhoud website / boek / app** | Vast programma van 10 weken | Losse onderdelen/oefeningen waar u uit kunt kiezen, doorlopend |
| **Inhoud oefeningen (zelfhulp)** | Zelfhulp gebaseerd op cognitieve gedragstherapie | Zelfhulp gebaseerd op probleem oplossende therapie |
| **Persoonlijk preventieplan** | Persoonlijk preventieplan is uitgangspunt van de behandeling | U heeft geen persoonlijk preventieplan |
| **Tijdsinvestering** | 1 uur per week | ½ uur per week |
| **Bescherming tegen terugval** | Uw risico op terugval neemt af van 60% naar 54% | Uw risico op terugval neemt af van 60% naar45% |

Stel u voor dat u deze behandeling krijgt aangeboden na het afsluiten van de behandeling bij de GGZ. Welke behandeling kiest u (kruis aan)?


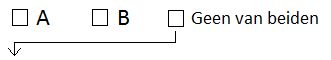


*(Wanneer u geen van beiden heeft ingevuld 🡪)* Als u moest kiezen, welke behandeling zou u dan kiezen?


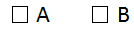


| **4.** | **Behandeling A** | **Behandeling B** |
| --- | --- | --- |
| **Gesprek met een behandelaar** | 1x per 3 maanden | 1x per 6 maanden |
| **Website, zelfhulpboek of app met informatie, oefeningen en tips om terugval te voorkómen** | Ondersteuning via App op iPad / mobiele telefoon | Ondersteuning via zelfhulpboek |
| **Inhoud website / boek / app** | Losse onderdelen/oefeningen waar u uit kunt kiezen, doorlopend | Vast programma van 10 weken |
| **Inhoud oefeningen (zelfhulp)** | Zelfhulp gebaseerd op mentale fitheid | Zelfhulp gebaseerd op cognitieve gedragstherapie |
| **Persoonlijk preventieplan** | U heeft geen persoonlijk preventieplan | Persoonlijk preventieplan is uitgangspunt van de behandeling |
| **Tijdsinvestering** | ½ uur per week | 1 uur per week |
| **Bescherming tegen terugval** | Uw risico op terugval neemt af van 60% naar 54% | Uw risico op terugval neemt af van 60% naar 45% |

Stel u voor dat u deze behandeling krijgt aangeboden na het afsluiten van de behandeling bij de GGZ. Welke behandeling kiest u (kruis aan)?


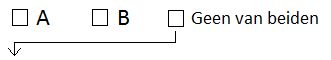


*(Wanneer u geen van beiden heeft ingevuld 🡪)* Als u moest kiezen, welke behandeling zou u dan kiezen?


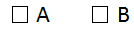


| **5.** | **Behandeling A** | **Behandeling B** |
| --- | --- | --- |
| **Gesprek met een behandelaar** | Alleen wanneer u weer klachten krijgt | 1x per 3 maanden |
| **Website, zelfhulpboek of app met informatie, oefeningen en tips om terugval te voorkómen** | Ondersteuning via App op iPad / mobiele telefoon | Ondersteuning via zelfhulpboek |
| **Inhoud website / boek / app** | Losse onderdelen/oefeningen waar u uit kunt kiezen, doorlopend | Vast programma van 10 weken |
| **Inhoud oefeningen (zelfhulp)** | Zelfhulp gebaseerd op mentale fitheid | Zelfhulp gebaseerd op cognitieve gedragstherapie |
| **Persoonlijk preventieplan** | U heeft geen persoonlijk preventieplan | Persoonlijk preventieplan is uitgangspunt van de behandeling |
| **Tijdsinvestering** | 1 uur per week | 2 uur per week |
| **Bescherming tegen terugval** | Uw risico op terugval neemt af van 60% naar 45% | Uw risico op terugval neemt af van 60% naar 36% |

Stel u voor dat u deze behandeling krijgt aangeboden na het afsluiten van de behandeling bij de GGZ. Welke behandeling kiest u (kruis aan)?


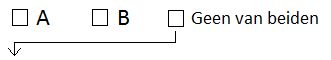


*(Wanneer u geen van beiden heeft ingevuld 🡪)* Als u moest kiezen, welke behandeling zou u dan kiezen?


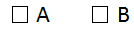


| **6.** | **Behandeling A** | **Behandeling B** |
| --- | --- | --- |
| **Gesprek met een behandelaar** | Alleen wanneer u weer klachten krijgt | 1x per 6 maanden |
| **Website, zelfhulpboek of app met informatie, oefeningen en tips om terugval te voorkómen** | Ondersteuning via website | Ondersteuning via App op iPad / mobiele telefoon |
| **Inhoud website / boek / app** | Losse onderdelen/oefeningen waar u uit kunt kiezen, doorlopend | Vast programma van 10 weken |
| **Inhoud oefeningen (zelfhulp)** | Zelfhulp gebaseerd op mindfulness | Zelfhulp gebaseerd op mentale fitheid |
| **Persoonlijk preventieplan** | Persoonlijk preventieplan is uitgangspunt van de behandeling | U heeft geen persoonlijk preventieplan |
| **Tijdsinvestering** | 1 uur per week | ½ uur per week |
| **Bescherming tegen terugval** | Uw risico op terugval neemt af van 60% naar 54% | Uw risico op terugval neemt af van 60% naar 45% |

Stel u voor dat u deze behandeling krijgt aangeboden na het afsluiten van de behandeling bij de GGZ. Welke behandeling kiest u (kruis aan)?


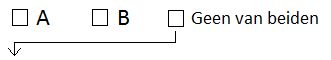


*(Wanneer u geen van beiden heeft ingevuld 🡪)* Als u moest kiezen, welke behandeling zou u dan kiezen?


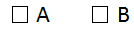


| **7.** | **Behandeling A** | **Behandeling B** |
| --- | --- | --- |
| **Gesprek met een behandelaar** | 1x per maand | Alleen wanneer u weer klachten krijgt |
| **Website, zelfhulpboek of app met informatie, oefeningen en tips om terugval te voorkómen** | Ondersteuning via website | Ondersteuning via App op iPad / mobiele telefoon |
| **Inhoud website / boek / app** | Vast programma van 10 weken | Losse onderdelen/oefeningen waar u uit kunt kiezen, doorlopend |
| **Inhoud oefeningen (zelfhulp)** | Zelfhulp gebaseerd op mindfulness | Zelfhulp gebaseerd op cognitieve gedragstherapie |
| **Persoonlijk preventieplan** | U heeft geen persoonlijk preventieplan | Persoonlijk preventieplan is uitgangspunt van de behandeling |
| **Tijdsinvestering** | 1 uur per week | 2 uur per week |
| **Bescherming tegen terugval** | Uw risico op terugval neemt af van 60% naar 54% | Uw risico op terugval neemt af van 60% naar 45% |

Stel u voor dat u deze behandeling krijgt aangeboden na het afsluiten van de behandeling bij de GGZ. Welke behandeling kiest u (kruis aan)?


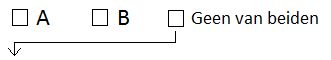


*(Wanneer u geen van beiden heeft ingevuld 🡪)* Als u moest kiezen, welke behandeling zou u dan kiezen?


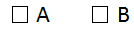


| **8.** | **Behandeling A** | **Behandeling B** |
| --- | --- | --- |
| **Gesprek met een behandelaar** | 1x per 6 maanden | 1x per maand |
| **Website, zelfhulpboek of app met informatie, oefeningen en tips om terugval te voorkómen** | Ondersteuning via App op iPad / mobiele telefoon | Ondersteuning via website |
| **Inhoud website / boek / app** | Vast programma van 10 weken | Losse onderdelen/oefeningen waar u uit kunt kiezen, doorlopend |
| **Inhoud oefeningen (zelfhulp)** | Zelfhulp gebaseerd op probleem oplossende therapie | Zelfhulp gebaseerd op mindfulness |
| **Persoonlijk preventieplan** | Persoonlijk preventieplan is uitgangspunt van de behandeling | U heeft geen persoonlijk preventieplan |
| **Tijdsinvestering** | ½ uur per week | 2 uur per week |
| **Bescherming tegen terugval** | Uw risico op terugval neemt af van 60% naar 54% | Uw risico op terugval neemt af van 60% naar 45% |

Stel u voor dat u deze behandeling krijgt aangeboden na het afsluiten van de behandeling bij de GGZ. Welke behandeling kiest u (kruis aan)?


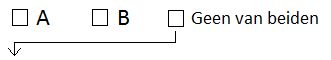


*(Wanneer u geen van beiden heeft ingevuld 🡪)* Als u moest kiezen, welke behandeling zou u dan kiezen?


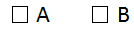


| **9.** | **Behandeling A** | **Behandeling B** |
| --- | --- | --- |
| **Gesprek met een behandelaar** | Alleen wanneer u weer klachten krijgt | 1x per 6 maanden |
| **Website, zelfhulpboek of app met informatie, oefeningen en tips om terugval te voorkómen** | Ondersteuning via App op iPad / mobiele telefoon | Ondersteuning via website |
| **Inhoud website / boek / app** | Vast programma van 10 weken | Losse onderdelen/oefeningen waar u uit kunt kiezen, doorlopend |
| **Inhoud oefeningen (zelfhulp)** | Zelfhulp gebaseerd op cognitieve gedragstherapie | Zelfhulp gebaseerd op mentale fitheid |
| **Persoonlijk preventieplan** | U heeft geen persoonlijk preventieplan | Persoonlijk preventieplan is uitgangspunt van de behandeling |
| **Tijdsinvestering** | 1 uur per week | 1 uur per week |
| **Bescherming tegen terugval** | Uw risico op terugval neemt af van 60% naar 54% | Uw risico op terugval neemt af van 60% naar 36% |

Stel u voor dat u deze behandeling krijgt aangeboden na het afsluiten van de behandeling bij de GGZ. Welke behandeling kiest u (kruis aan)?


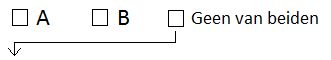


*(Wanneer u geen van beiden heeft ingevuld 🡪)* Als u moest kiezen, welke behandeling zou u dan kiezen?


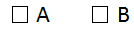


| **10.** | **Behandeling A** | **Behandeling B** |
| --- | --- | --- |
| **Gesprek met een behandelaar** | 1x per maand | 1x per 3 maanden |
| **Website, zelfhulpboek of app met informatie, oefeningen en tips om terugval te voorkómen** | Ondersteuning via website | Ondersteuning via App op iPad / mobiele telefoon |
| **Inhoud website / boek / app** | Losse onderdelen/oefeningen waar u uit kunt kiezen, doorlopend | Vast programma van 10 weken |
| **Inhoud oefeningen (zelfhulp)** | Zelfhulp gebaseerd op mentale fitheid | Zelfhulp gebaseerd op mindfulness |
| **Persoonlijk preventieplan** | Persoonlijk preventieplan is uitgangspunt van de behandeling | U heeft geen persoonlijk preventieplan |
| **Tijdsinvestering** | ½ uur per week | 2 uur per week |
| **Bescherming tegen terugval** | Uw risico op terugval neemt af van 60% naar 45% | Uw risico op terugval neemt af van 60% naar 36% |

Stel u voor dat u deze behandeling krijgt aangeboden na het afsluiten van de behandeling bij de GGZ. Welke behandeling kiest u (kruis aan)?


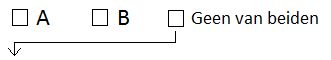


*(Wanneer u geen van beiden heeft ingevuld 🡪)* Als u moest kiezen, welke behandeling zou u dan kiezen?


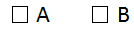


| **11.** | **Behandeling A** | **Behandeling B** |
| --- | --- | --- |
| **Gesprek met een behandelaar** | 1x per 3 maanden | Alleen wanneer u weer klachten krijgt |
| **Website, zelfhulpboek of app met informatie, oefeningen en tips om terugval te voorkómen** | Ondersteuning via website | Ondersteuning via zelfhulpboek |
| **Inhoud website / boek / app** | Losse onderdelen/oefeningen waar u uit kunt kiezen, doorlopend | Vast programma van 10 weken |
| **Inhoud oefeningen (zelfhulp)** | Zelfhulp gebaseerd op probleem oplossende therapie | Zelfhulp gebaseerd op mindfulness |
| **Persoonlijk preventieplan** | Persoonlijk preventieplan is uitgangspunt van de behandeling | U heeft geen persoonlijk preventieplan |
| **Tijdsinvestering** | 2 uur per week | 1 uur per week |
| **Bescherming tegen terugval** | Uw risico op terugval neemt af van 60% naar 36% | Uw risico op terugval neemt af van 60% naar 54% |

Stel u voor dat u deze behandeling krijgt aangeboden na het afsluiten van de behandeling bij de GGZ. Welke behandeling kiest u (kruis aan)?


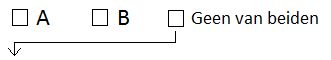


*(Wanneer u geen van beiden heeft ingevuld 🡪)* Als u moest kiezen, welke behandeling zou u dan kiezen?


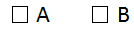


| **12.** | **Behandeling A** | **Behandeling B** |
| --- | --- | --- |
| **Gesprek met een behandelaar** | 1x per 6 maanden | 1x per 3 maanden |
| **Website, zelfhulpboek of app met informatie, oefeningen en tips om terugval te voorkómen** | Ondersteuning via zelfhulpboek | Ondersteuning via website |
| **Inhoud website / boek / app** | Losse onderdelen/oefeningen waar u uit kunt kiezen, doorlopend | Vast programma van 10 weken |
| **Inhoud oefeningen (zelfhulp)** | Zelfhulp gebaseerd op mentale fitheid | Zelfhulp gebaseerd op probleem oplossende therapie |
| **Persoonlijk preventieplan** | Persoonlijk preventieplan is uitgangspunt van de behandeling | U heeft geen persoonlijk preventieplan |
| **Tijdsinvestering** | 2 uur per week | 1 uur per week |
| **Bescherming tegen terugval** | Uw risico op terugval neemt af van 60% naar 36% | Uw risico op terugval neemt af van 60% naar 45% |

Stel u voor dat u deze behandeling krijgt aangeboden na het afsluiten van de behandeling bij de GGZ. Welke behandeling kiest u (kruis aan)?


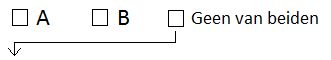


*(Wanneer u geen van beiden heeft ingevuld 🡪)* Als u moest kiezen, welke behandeling zou u dan kiezen?


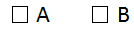


| **13.** | **Behandeling A** | **Behandeling B** |
| --- | --- | --- |
| **Gesprek met een behandelaar** | Alleen wanneer u weer klachten krijgt | 1x per 6 maanden |
| **Website, zelfhulpboek of app met informatie, oefeningen en tips om terugval te voorkómen** | Ondersteuning via App op iPad / mobiele telefoon | Ondersteuning via website |
| **Inhoud website / boek / app** | Vast programma van 10 weken | Losse onderdelen/oefeningen waar u uit kunt kiezen, doorlopend |
| **Inhoud oefeningen (zelfhulp)** | Zelfhulp gebaseerd op probleem oplossende therapie | Zelfhulp gebaseerd op cognitieve gedragstherapie |
| **Persoonlijk preventieplan** | Persoonlijk preventieplan is uitgangspunt van de behandeling | U heeft geen persoonlijk preventieplan |
| **Tijdsinvestering** | 1 uur per week | ½ uur per week |
| **Bescherming tegen terugval** | Uw risico op terugval neemt af van 60% naar 36% | Uw risico op terugval neemt af van 60% naar 54% |

Stel u voor dat u deze behandeling krijgt aangeboden na het afsluiten van de behandeling bij de GGZ. Welke behandeling kiest u (kruis aan)?


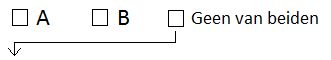


*(Wanneer u geen van beiden heeft ingevuld 🡪)* Als u moest kiezen, welke behandeling zou u dan kiezen?


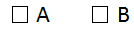


| **14.** | **Behandeling A** | **Behandeling B** |
| --- | --- | --- |
| **Gesprek met een behandelaar** | 1x per 6 maanden | 1x per maand |
| **Website, zelfhulpboek of app met informatie, oefeningen en tips om terugval te voorkómen** | Ondersteuning via zelfhulpboek | Ondersteuning via App op iPad / mobiele telefoon |
| **Inhoud website / boek / app** | Losse onderdelen/oefeningen waar u uit kunt kiezen, doorlopend | Vast programma van 10 weken |
| **Inhoud oefeningen (zelfhulp)** | Zelfhulp gebaseerd op mindfulness | Zelfhulp gebaseerd op mentale fitheid |
| **Persoonlijk preventieplan** | U heeft geen persoonlijk preventieplan | U heeft geen persoonlijk preventieplan |
| **Tijdsinvestering** | 1 uur per week | 2 uur per week |
| **Bescherming tegen terugval** | Uw risico op terugval neemt af van 60% naar 36% | Uw risico op terugval neemt af van 60% naar 45% |

Stel u voor dat u deze behandeling krijgt aangeboden na het afsluiten van de behandeling bij de GGZ. Welke behandeling kiest u (kruis aan)?


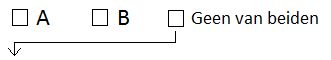


*(Wanneer u geen van beiden heeft ingevuld 🡪)* Als u moest kiezen, welke behandeling zou u dan kiezen?


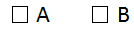


| **15.** | **Behandeling A** | **Behandeling B** |
| --- | --- | --- |
| **Gesprek met een behandelaar** | 1x per maand | 1x per 3 maanden |
| **Website, zelfhulpboek of app met informatie, oefeningen en tips om terugval te voorkómen** | Ondersteuning via zelfhulpboek | Ondersteuning via App op iPad / mobiele telefoon |
| **Inhoud website / boek / app** | Losse onderdelen/oefeningen waar u uit kunt kiezen, doorlopend | Vast programma van 10 weken |
| **Inhoud oefeningen (zelfhulp)** | Zelfhulp gebaseerd op probleem oplossende therapie | Zelfhulp gebaseerd op mindfulness |
| **Persoonlijk preventieplan** | U heeft geen persoonlijk preventieplan | Persoonlijk preventieplan is uitgangspunt van de behandeling |
| **Tijdsinvestering** | 2 uur per week | ½ uur per week |
| **Bescherming tegen terugval** | Uw risico op terugval neemt af van 60% naar 36% | Uw risico op terugval neemt af van 60% naar 45% |

Stel u voor dat u deze behandeling krijgt aangeboden na het afsluiten van de behandeling bij de GGZ. Welke behandeling kiest u (kruis aan)?


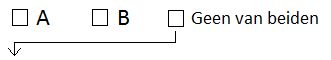


*(Wanneer u geen van beiden heeft ingevuld 🡪)* Als u moest kiezen, welke behandeling zou u dan kiezen?


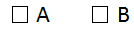


| **16.** | **Behandeling A** | **Behandeling B** |
| --- | --- | --- |
| **Gesprek met een behandelaar** | 1x per maand | Alleen wanneer u weer klachten krijgt |
| **Website, zelfhulpboek of app met informatie, oefeningen en tips om terugval te voorkómen** | Ondersteuning via zelfhulpboek | Ondersteuning via website |
| **Inhoud website / boek / app** | Vast programma van 10 weken | Losse onderdelen/oefeningen waar u uit kunt kiezen, doorlopend |
| **Inhoud oefeningen (zelfhulp)** | Zelfhulp gebaseerd op probleem oplossende therapie | Zelfhulp gebaseerd op mentale fitheid |
| **Persoonlijk preventieplan** | Persoonlijk preventieplan is uitgangspunt van de behandeling | Persoonlijk preventieplan is uitgangspunt van de behandeling |
| **Tijdsinvestering** | ½ uur per week | 1 uur per week |
| **Bescherming tegen terugval** | Uw risico op terugval neemt af van 60% naar 45% | Uw risico op terugval neemt af van 60% naar 54% |

Stel u voor dat u deze behandeling krijgt aangeboden na het afsluiten van de behandeling bij de GGZ. Welke behandeling kiest u (kruis aan)?


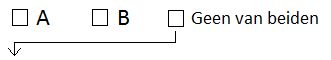


*(Wanneer u geen van beiden heeft ingevuld 🡪)* Als u moest kiezen, welke behandeling zou u dan kiezen?


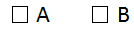


| **17.** | **Behandeling A** | **Behandeling B** |
| --- | --- | --- |
| **Gesprek met een behandelaar** | 1x per 3 maanden | 1x per maand |
| **Website, zelfhulpboek of app met informatie, oefeningen en tips om terugval te voorkómen** | Ondersteuning via zelfhulpboek | Ondersteuning via App op iPad / mobiele telefoon |
| **Inhoud website / boek / app** | Vast programma van 10 weken | Losse onderdelen/oefeningen waar u uit kunt kiezen, doorlopend |
| **Inhoud oefeningen (zelfhulp)** | Zelfhulp gebaseerd op cognitieve gedragstherapie | Zelfhulp gebaseerd op mindfulness |
| **Persoonlijk preventieplan** | U heeft geen persoonlijk preventieplan | Persoonlijk preventieplan is uitgangspunt van de behandeling |
| **Tijdsinvestering** | 1 uur per week | 2 uur per week |
| **Bescherming tegen terugval** | Uw risico op terugval neemt af van 60% naar 45% | Uw risico op terugval neemt af van 60% naar 36% |

Stel u voor dat u deze behandeling krijgt aangeboden na het afsluiten van de behandeling bij de GGZ. Welke behandeling kiest u (kruis aan)?


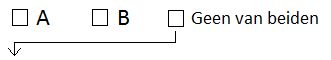


*(Wanneer u geen van beiden heeft ingevuld 🡪)* Als u moest kiezen, welke behandeling zou u dan kiezen?


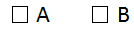


| **18.** | **Behandeling A** | **Behandeling B** |
| --- | --- | --- |
| **Gesprek met een behandelaar** | 1x per 6 maanden | 1x per maand |
| **Website, zelfhulpboek of app met informatie, oefeningen en tips om terugval te voorkómen** | Ondersteuning via website | Ondersteuning via zelfhulpboek |
| **Inhoud website / boek / app** | Vast programma van 10 weken | Losse onderdelen/oefeningen waar u uit kunt kiezen, doorlopend |
| **Inhoud oefeningen (zelfhulp)** | Zelfhulp gebaseerd op mindfulness | Zelfhulp gebaseerd op cognitieve gedragstherapie |
| **Persoonlijk preventieplan** | Persoonlijk preventieplan is uitgangspunt van de behandeling | U heeft geen persoonlijk preventieplan |
| **Tijdsinvestering** | 2 uur per week | ½ uur per week |
| **Bescherming tegen terugval** | Uw risico op terugval neemt af van 60% naar 45% | Uw risico op terugval neemt af van 60% naar 54% |

Stel u voor dat u deze behandeling krijgt aangeboden na het afsluiten van de behandeling bij de GGZ. Welke behandeling kiest u (kruis aan)?


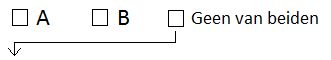


*(Wanneer u geen van beiden heeft ingevuld 🡪)* Als u moest kiezen, welke behandeling zou u dan kiezen?


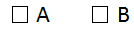


| **19.** | **Behandeling A** | **Behandeling B** |
| --- | --- | --- |
| **Gesprek met een behandelaar** | 1x per maand | Alleen wanneer u weer klachten krijgt |
| **Website, zelfhulpboek of app met informatie, oefeningen en tips om terugval te voorkómen** | Ondersteuning via App op iPad / mobiele telefoon | Ondersteuning via zelfhulpboek |
| **Inhoud website / boek / app** | Losse onderdelen/oefeningen waar u uit kunt kiezen, doorlopend | Vast programma van 10 weken |
| **Inhoud oefeningen (zelfhulp)** | Zelfhulp gebaseerd op cognitieve gedragstherapie | Zelfhulp gebaseerd op probleem oplossende therapie |
| **Persoonlijk preventieplan** | Persoonlijk preventieplan is uitgangspunt van de behandeling | U heeft geen persoonlijk preventieplan |
| **Tijdsinvestering** | 1 uur per week | ½ uur per week |
| **Bescherming tegen terugval** | Uw risico op terugval neemt af van 60% naar 36% | Uw risico op terugval neemt af van 60% naar 54% |

Stel u voor dat u deze behandeling krijgt aangeboden na het afsluiten van de behandeling bij de GGZ. Welke behandeling kiest u (kruis aan)?


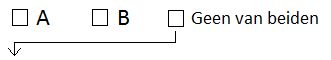


*(Wanneer u geen van beiden heeft ingevuld 🡪)* Als u moest kiezen, welke behandeling zou u dan kiezen?


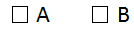


| **20.** | **Behandeling A** | **Behandeling B** |
| --- | --- | --- |
| **Gesprek met een behandelaar** | 1x per 3 maanden | 1x per maand |
| **Website, zelfhulpboek of app met informatie, oefeningen en tips om terugval te voorkómen** | Ondersteuning via zelfhulpboek | Ondersteuning via website |
| **Inhoud website / boek / app** | Losse onderdelen/oefeningen waar u uit kunt kiezen, doorlopend | Vast programma van 10 weken |
| **Inhoud oefeningen (zelfhulp)** | Zelfhulp gebaseerd op mindfulness | Zelfhulp gebaseerd op probleem oplossende therapie |
| **Persoonlijk preventieplan** | U heeft geen persoonlijk preventieplan | Persoonlijk preventieplan is uitgangspunt van de behandeling |
| **Tijdsinvestering** | ½ uur per week | 1 uur per week |
| **Bescherming tegen terugval** | Uw risico op terugval neemt af van 60% naar 45% | Uw risico op terugval neemt af van 60% naar 54% |

Stel u voor dat u deze behandeling krijgt aangeboden na het afsluiten van de behandeling bij de GGZ. Welke behandeling kiest u (kruis aan)?


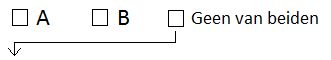


*(Wanneer u geen van beiden heeft ingevuld 🡪)* Als u moest kiezen, welke behandeling zou u dan kiezen?


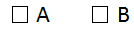


Heeft u op- of aanmerkingen over deze vragenlijst? Dan kunt u dit hieronder aangeven.

**Gaat u alstublieft op de volgende pagina verder met het laatste deel van de vragenlijst. Deze vragen gaan over uw gezondheid, uw klachten en uw functioneren.**

[A. WHO Disability Assessment Schedule (WHODAS) [50]]

[B. Inventory of Depressive Symptomatology (IDS) [51]]

[C. Beck Anxiety Inventory (BAI) [52]]

[D. Anxiety Sensitivity Index (ASI) [53]]
