# Supplementary material for: Patient preferences for a guided self-help programme to prevent relapse in anxiety or depression: A discrete choice experiment
Source: PLoS One. 2019 Jul 18;14(7):e0219588. doi: 10.1371/journal.pone.0219588 (PMC6638925; doi:10.1371/journal.pone.0219588)
Supplement: S6 File — (DOC) [file pone.0219588.s009.doc]

**Vragenlijst**

De volgende vragen gaan over uw voorkeur voor een onderhoudsbehandeling nadat u de behandeling bij GGZ inGeest heeft afgerond. Deze behandeling bestaat nu nog niet, maar wij gaan deze in de toekomst wel aanbieden. De onderhoudsbehandeling kan uit verschillende onderdelen bestaan.

**Kunt u bij elk van de volgende onderdelen aangeven hoe belangrijk u deze vindt als onderdeel van een onderhoudsbehandeling na afronden van uw behandeling bij GGZ inGeest?**

| **Na afronding van mijn behandeling bij GGZ inGeest is het voor mij belangrijk dat…..** | Helemaal niet belang-rijk | Niet belang-rijk | Neutraal | Wel belang-rijk | Heel belangrijk |
| --- | --- | --- | --- | --- | --- |
| ….ik een vaste behandelaar heb met wie ik contact op kan nemen | 1 | 2 | 3 | 4 | 5 |
| ….ik zelf kan bepalen hoeveel gesprekken per jaar ik nodig heb | 1 | 2 | 3 | 4 | 5 |
| ….ik de mogelijkheid heb om via email contact op te nemen met een behandelaar | 1 | 2 | 3 | 4 | 5 |
| ….mijn nieuwe behandelaar op de hoogte is van wat ik in de behandeling heb geleerd | 1 | 2 | 3 | 4 | 5 |

| **Na afronding van mijn behandeling bij GGZ inGeest zou ik graag beschikking hebben over….** | Helemaal oneens | Oneens | Neutraal | Eens | Helemaal eens |
| --- | --- | --- | --- | --- | --- |
| …..een applicatie op mijn smartphone met informatie, oefeningen en tips om terugval te voorkómen | 1 | 2 | 3 | 4 | 5 |
| ….een zelfhulpboek met informatie, oefeningen en tips om terugval te voorkómen | 1 | 2 | 3 | 4 | 5 |
| ….een website met informatie, oefeningen en tips om terugval te voorkómen | 1 | 2 | 3 | 4 | 5 |

| **Mijn keuze om gebruik te maken van een onderhoudsbehandeling hangt af van….** | Helemaal oneens | Oneens | Neutraal | Eens | Helemaal eens |
| --- | --- | --- | --- | --- | --- |
| …….hoeveel tijd ik moet besteden aan de onderhoudsbehandeling | 1 | 2 | 3 | 4 | 5 |
| …..hoe effectief de behandeling is in het verminderen van mijn risico op terugval | 1 | 2 | 3 | 4 | 5 |
| ….of mijn zorgverzekeraar de kosten van de behandeling vergoedt | 1 | 2 | 3 | 4 | 5 |

| **Ik zou eerder gebruik maken van een website ter ondersteuning als….** | Helemaal oneens | Oneens | Neutraal | Eens | Helemaal eens |
| --- | --- | --- | --- | --- | --- |
| …..ik op de website oefeningen kan doen die mij helpen om wat ik geleerd heb in de therapie vast te houden | 1 | 2 | 3 | 4 | 5 |
| …mijn behandelaar mij ondersteunt bij het gebruiken van de website | 1 | 2 | 3 | 4 | 5 |
| …mijn behandelaar feedback geeft op de oefeningen die ik via de website maak | 1 | 2 | 3 | 4 | 5 |
| ….ik een persoonlijk plan kan maken op de website om terugval te voorkómen (terugval-preventie-plan) | 1 | 2 | 3 | 4 | 5 |
| …de oefeningen op de website mij helpen om problemen te leren oplossen | 1 | 2 | 3 | 4 | 5 |
| …de oefeningen op de website mij helpen om mijn negatieve gedachten te veranderen en mijn gedrag aan te passen | 1 | 2 | 3 | 4 | 5 |
| ……de oefeningen op de website mij helpen om in het hier en nu te leven | 1 | 2 | 3 | 4 | 5 |
| …de oefeningen op de website mij helpen om mijn leven richting te geven | 1 | 2 | 3 | 4 | 5 |
| …de website een vast programma volgt dat bestaat uit een aantal lessen | 1 | 2 | 3 | 4 | 5 |
| ….de website veel verschillende oefeningen en cursussen heeft waar ik uit kan kiezen | 1 | 2 | 3 | 4 | 5 |
| …ik herinnerd word om te oefenen per sms of e-mail | 1 | 2 | 3 | 4 | 5 |
| …ik kan chatten met andere (ex-) patiënten | 1 | 2 | 3 | 4 | 5 |
| …ik via de website contact op kan nemen met een psychiater | 1 | 2 | 3 | 4 | 5 |
| ….ik de website ook via mijn tablet kan gebruiken | 1 | 2 | 3 | 4 | 5 |
| ….de website mooi is vormgegeven | 1 | 2 | 3 | 4 | 5 |
| ...de website gemakkelijk te gebruiken is | 1 | 2 | 3 | 4 | 5 |
| …de website overzichtelijk is | 1 | 2 | 3 | 4 | 5 |
| ….ik informatie en gemaakte oefeningen kan downloaden | 1 | 2 | 3 | 4 | 5 |
| …ik informatie van de website kan delen met familie/vrienden/hulpverleners | 1 | 2 | 3 | 4 | 5 |
| …ik zelf kan bijhouden of mijn klachten weer erger worden | 1 | 2 | 3 | 4 | 5 |

| **Wat verder belangrijk is in mijn keuze om gebruik te maken van een onderhoudsbehandeling…. (vul in)** |
| --- |
|  |
|  |
|  |
|  |
|  |
